# Impact of Rural Trauma Team Development Education on Prehospital Time, Referral-to-Dispatch Interval, and Neurological and Musculoskeletal Injury Outcomes: Cluster Randomized Controlled Trial

**DOI:** 10.2196/82591

**Published:** 2026-04-20

**Authors:** Herman Lule, Micheal Mugerwa, Anne Abio, Benson Oguttu, Andrew Kakeeto, Fiona J Walsh, Hervé Monka Lekuya, Robinson Ssebuufu, Patrick Kyamanywa, Andreas Deckert, Till Bärnighausen, Jussi P Posti, Michael Lowery Wilson

**Affiliations:** 1 Injury Epidemiology and Prevention (IEP) Research Group, Turku Brain Injury Centre, Division of Clinical Neurosciences, Turku University Hospital and University of Turku, Vähä Hämeenkatu 1B, Turun Yliopisto, Turku, FI20500, Finland, 358 465699821, 023132737; 2 Center for Health Equity in Surgery and Anesthesia, Institute of Global Health Sciences, University of California, San Francisco, CA, United States; 3 Department of Surgery, Kiryandongo Regional Referral Hospital, Kigumba, Uganda; 4 Department of Public Health Sciences, California Baptist University, Riverside, CA, United States; 5 Research Centre for Child Psychiatry, University of Turku, Turku, Finland; 6 INVEST Research Flagship Centre, University of Turku, Turku, Finland; 7 Department of Surgery, Jinja Regional Referral Hospital, Jinja, Uganda; 8 Department of Public Health, Botswana-Havard Health Partnership (BHP), Gaborone, Botswana; 9 Department of Surgery, Hoima Regional Referral Hospital, Hoima, Uganda; 10 Department of Surgery, Kampala International University, Kampala, Uganda; 11 Heidelberg Institute of Global Health, Heidelberg University, Heidelberg, Baden-Wurttemberg, Germany; 12 Department of Neurosurgery, College of Health Sciences, Makerere University, Kampala, Uganda; 13 Department of Human Structure and Repair (GE38), Faculty of Medicine, Ghent University, Flanders, Belgium; 14 Department of Surgery, Mengo Hospital, Kampala, Uganda; 15 Mother Kevin Postgraduate Medical School, Uganda Martyr's University, Nkozi, Uganda; 16 Africa Health Research Institute, Durban, South Africa; 17 Department of Global Health and Population, Havard T H Chan School of Public Health, Havard University, Boston, MA, United States; 18 Neurocenter, Department of Neurosurgery and Turku Brain Injury Center, Turku University Hospital and University of Turku, Turku, Finland

**Keywords:** trauma, education, training, prehospital, outcomes, neurology, musculoskeletal, injuries, cluster randomized trial

## Abstract

**Background:**

Scarce human resources for health and high injury-related mortality coincide with inequities in accessing quality trauma education programs in low- and middle-income countries. Existing observational studies restrict assessments of trauma training program impacts on providers’ knowledge. Evaluation of trauma education programs outside clinical trial settings hinders their effectiveness in influencing clinical practice and policy changes for patient outcomes.

**Objective:**

This study aimed to assess the impact of the Rural Trauma Team Development Course (RTTDC) on clinical processes and patient outcomes of motorcycle-accident–related neurological and/or musculoskeletal injuries in selected Ugandan hospitals.

**Methods:**

This was a pragmatic 2-arm, parallel, multiperiod, cluster randomized controlled trial. The participants were trauma care frontline personnel and patients aged 2‐80 years at 3 intervention and 3 control Ugandan hospitals (1:1 allocation). Hospitals were randomly allocated to intervention or control groups using permuted block sequences. Sequence codes were generated off-site by an independent statistician using Sealed Envelope (version 1.23.1; Sealed Envelope Ltd). Both patient participants and outcome assessors were blinded to allocation. Hospital allocation codes were concealed until the point of assignment. In the intervention arm, 500 trauma care frontliners received RTTDC, whereas patients received standard care. In the control arm, patients received standard care without RTTDC for staff. The primary outcomes were time from accident to admission and from referral to dispatch. The secondary outcomes were all-cause 90-day mortality and morbidity related to neurological and/or musculoskeletal injuries. We followed the CONSORT (Consolidated Standards of Reporting Trials) guidelines for reporting cluster randomized trials.

**Results:**

We analyzed 1003 participants (501 intervention and 502 control). The intervention arm had a shorter median (IQR) prehospital time of 1 hour (0.50‐2) and referral-to-dispatch interval during interfacility transfers of 2 hours (1.25‐2.75). This contrasted with 2 hours (1.50‐4) and 4 hours (2.50‐4.10) in the control arm, respectively *(P*<.001). The 90-day mortality was more than halved in the intervention (5%, 24/457) vs in the control arm (13%, 58/430) *(P*<.001). Fewer participants in the intervention group had unfavorable Glasgow Outcome Scale scores (9%, 42/457) vs (20%, 87/430) *(P*<.001). No difference was found in musculoskeletal injury morbidity outcomes *(P*=.57).

**Conclusions:**

Rural trauma team development training demonstrated potential for improved organizational time efficiency and clinical outcomes for neurological injuries without negatively impacting musculoskeletal injury morbidity outcomes. Evidence from this trial supports that locally contextualized, trainee-led rural trauma team development interventional programs are feasible in low- and middle-income countries. However, despite being a multicenter study conducted across 6 geographically distinct hospitals, the research is limited in generalizability due to its focus on a single health care system within 1 country, retrospective trial registration, exclusion of prehospital deaths, and a relatively small number of clusters, which could introduce measurement bias.

## Introduction

### Background

Globally, access to essential surgical services and trauma education remains restricted. In Africa, shortages of health care workers and their inequitable distribution further impede access to both emergency and elective surgical care. As a result, a substantial proportion of the population in Africa lacks access to these critical services [1].

Optimizing human resource capacity, particularly for trauma care, is critical for strengthening health care systems, alongside infrastructure development [2]. The provision of trauma education for nonspecialist primary care providers remains a potential remedy to address the shortage of health care human resources in Africa [3], but linking such medical education programs to patient outcomes has been underresearched. While several training programs such as the Advanced Trauma Life Support (ATLS) and Primary Trauma Course (PTC) have been introduced, financial barriers and limited assessment of the impact on patient outcomes have hindered the implementation of these education programs, particularly for frontline trauma care providers in low- and middle-income countries (LMICs), who include lay response personnel such as traffic police [3,4]. According to a scoping review of trauma training courses, the average cost of ATLS per participant was US $820 compared to PTC (US $232) and Rural Trauma Team Development Course (RTTDC; US 50‐US $100) [5].

Locally contextualized trauma education programs are needed in LMICs due to resource constraints limiting ATLS uptake [5]. Other existing courses, such as PTC, face criticism for limited international recognition, a relative bias toward trauma technical skills, which limit lay responders’ participation, and their impact on patient outcomes has not been rigorously evaluated through clinical trials, warranting further exploration of need-specific alternatives [4].

Evidence from observational studies in the United States suggests that the RTTDC is a well-recognized form of trauma education. RTTDC can be flexibly implemented through a single-day format or a modular structure over approximately 8 hours, with instructors who are trauma specialists, nurses, or paramedics [6]. RTTDC emphasizes technical skills, including primary and secondary surveys, general trauma care, including for specific populations such as children and older adults, while also highlighting a team approach to injury management in rural environments. This is accomplished by evaluating team performance through simulations, didactic lectures, and case scenarios that focus on local injury burden and trauma nontechnical skills such as leadership, communication, coordination, resource management, and decision-making for interfacility transfers [6-8]. These components are vital for trauma teams in rural areas with limited human and infrastructural resources, enabling participation from nontechnical staff and connecting communities to formal trauma systems. Emphasizing trauma coordination, performance improvement, and situational resource awareness helps minimize delays in interfacility transfers for definitive care of injuries that exceed local capacity. While ATLS and PTC provide comprehensive approaches to technical trauma care, RTTDC offers a cost-effective option for training larger groups, including nontechnical staff, in resource-limited settings [5].

The ability of the RTTDC model to improve each trauma team member’s performance makes it applicable in LMICs, where team and organizational efficiency are more desirable to mitigate understaffing. However, researchers have primarily evaluated the course’s impact on provider performance rather than patient outcomes, as ethical constraints have precluded the evaluation of RTTDC interventions through individually randomized prospective clinical trials [8]. Furthermore, the need to mitigate potential contamination between treatment arms necessitates a cluster-randomized study design. Evaluation of surgical interventions and trauma education programs outside of randomized controlled settings contributes to research implementation gaps, limiting their utility in clinical practice [9]. Cumulative research findings from emerging contemporaneous studies are necessary to drive practice and policy changes [9].

While prior systematic reviews have identified improvements in clinician knowledge from trauma education programs in LMICs, the next critical step is to evaluate their impact on organizational efficiency and patient outcomes in the clinical setting [3-5]. Therefore, in this cluster-randomized trial, we examined the impact of a locally contextualized RTTDC on hospital processes and clinical outcomes in an LMIC setting, focusing on time efficiency, mortality, and morbidity of motorcycle accident–related neurological and/or musculoskeletal injuries. These injuries were previously identified as critical for strengthening rural trauma systems in Uganda [10]. Motorcycle-related crashes are the leading cause of fatal trauma in Uganda, and the resultant injuries are predominantly neurological or musculoskeletal in 85% of cases [11]. An injury was deemed motorcycle-related if it involved a motorcycle-motorcycle, motorcycle-pedestrian, motorcycle-vehicle, or motorcycle-static object crash. Motorcycle-related injury mechanism was established during the secondary survey in the evaluation of events surrounding the injury.

This study defined rural trauma team development education as the implementation of RTTDC and the establishment of trauma teams in rural Uganda, which were previously nonexistent. In this study, RTTDC was locally contextualized to fit within the interconnected geopolitical, socioeconomic, and health care system challenges in Uganda, and to conform to the 4 domains of the Kirkpatrick framework for evaluating educational interventions [12]. First, we acknowledged the constraints of limited human resources for health in low-income Uganda, political tensions, and internal conflicts; thus, medical interns and traffic law enforcement professionals formed an integral component of our frontline trauma team participants. These cadres provide the first contact to patients with injury in Uganda due to a weak, inadequately funded prehospital and emergency postcrash care system, largely resulting from a shift of resources to the high infectious disease burden and obstetric emergencies [13].

Second, through engagement with local surgical and medical education experts (university medical faculty deans) and the Ugandan Ministry of Health, we fitted our trauma education intervention within the existing framework of medical curricula. We concurrently timed the recruitment of study participants during medical internship placements and surgical clinical rotations to maximize the course uptake.

Third, we assessed participants’ satisfaction regarding the course through self-administered surveys (reaction), determined the effect of RTTDC intervention on participants’ knowledge through pre-post training trauma-based multiple-choice questions (knowledge gain and retention), and assessed the change in practice using the pre-post training trauma nontechnical skills scale based on customized trauma case scenarios that suited the local injury burden (behavioral change). The impact of RTTDC on participants’ knowledge, skills, and behavior was published elsewhere [14].

Finally, we translated classroom knowledge into clinical practice. We used the RTTDC participants to form well-coordinated, real-world rural trauma teams rooted at subcounties, using trained regional traffic police officers as the focal contact persons at the community level, and trained surgical residents at regional referral hospitals. Continuous reinforcement of RTTDC concepts to address team challenges was attained through weekly clinical audits with the trauma teams via Zoom (Zoom Video Communications, Inc). Consequently, these teams were involved in the development of a pilot Motorcycle Trauma Outcome Registry (MOTOR) using comprehensively pretested, refined, and locally customized quality indicators and measurable outcomes based on feedback from patient-public engagements. The data from the MOTOR registry were used in this study to directly link a trauma education intervention to patient outcomes through a trauma registry, thus completing the fourth level of the Kirkpatrick framework for rigorous evaluation of the effectiveness of RTTDC. The trial reporting followed the CONSORT (Consolidated Standards of Reporting Trials) guidelines for cluster-randomized trials, and the exploratory ancillary analyses due to COVID-19 interruptions adhered to the CONSORT and SPIRIT Extension for Randomized Controlled Trials Revised in Extenuating Circumstances (CONSERVE) guidelines [15,16].

### Null Hypothesis

We based the specific objectives of this trial on the null hypothesis that RTTDC training has no effect on the clinical process efficiency and outcomes of neurological and musculoskeletal injuries at both the treatment arm and individual levels.

### Specific Objectives

The study aimed to (1) determine the effect of RTTDC training on crash-to-admission and referral decision-to-dispatch intervals during interfacility transfers (primary outcomes); and (2) determine the effect of RTTDC training on all-cause 90-day mortality and morbidity of neurological and musculoskeletal injuries (secondary outcomes).

## Methods

### Overview

This was a pragmatic 2-arm, parallel, multiperiod, cluster randomized trial conducted from 9/01/2019 to 08/27/2023, with a 1-year suspension from 03/01/2020 to 03/01/2021 due to the extenuating effects of the COVID-19 pandemic. Six (6/17, 35%) Ugandan rural regional referral and teaching hospitals functioning as Level III trauma centers, including Jinja, Hoima, Fort Portal, Mubende, Kiryandongo, and Kampala International University Teaching Hospital, were included. Together, these hospitals serve a total population of 18 million people in 3 cities and 50 rural districts. Following the national standard of care, all patients requiring referral were transferred to the Level I trauma center, Mulago National Referral Hospital, Uganda’s comprehensive resource for multidisciplinary tertiary trauma care and rehabilitation.

Trauma centers are categorized into 5 levels based on their capabilities, with Levels IV and V being the lowest, providing initial stabilization through basic emergency facilities before transferring patients to higher-level centers. These correspond to health centers III and IV in Uganda. Levels III and II function as intermediate centers, offering 24/7 emergency assessment, resuscitation, and initiation of definitive care, including emergency surgeries, under the supervision of specialist surgeons, anesthesiologists, and radiologists, along with ongoing education programs for staff. In Uganda, these are typically represented by district hospitals and regional referral hospitals, respectively. While regional referral hospitals host surgical residency and internship programs to address human resource needs, they operate at Level III due to trauma resource challenges. Level I is a tertiary, superspecialized referral center that provides comprehensive care for severely injured patients, including complex microvascular surgeries, rehabilitation, and conducts trauma research, education, and quality assessment programs.

### Randomization and Masking

Hospitals (clusters) were randomized in a 1:1 ratio to the intervention or control arm using permuted-block randomization, assuming equal cluster sizes. Blocked randomization lists with 6 random codes were generated off-site by an independent statistician using Sealed Envelope (version 1.23.1; Sealed Envelope Ltd) [17]. Hospital allocation codes were concealed until the point of assignment; local hospital administrators enrolled staff after allocation, and patient recruitment began thereafter.

In the intervention arm (comprising 3 hospitals), 500 trauma care frontline personnel received the fourth edition of the American College of Surgeons RTTDC training, which was administered in a multiperiod manner, whereas in the control arm (3 hospitals), trauma care frontline personnel did not receive the training [6,18]. The training intervention was delivered by a specialist surgeon and 2 senior surgical residents. Outcome assessors were external medical officers or senior residents who were not involved in training delivery and who remained masked to hospital allocation by using deidentified records; patients were not informed of hospital allocation. The training status of specific providers was not revealed to outcome assessors.

### Eligibility Criteria

To be eligible to participate, a trauma center had to be a rural teaching hospital offering 24/7 emergency surgical care with access to blood banks and imaging, such as computed tomography (CT), X-ray, and ultrasound scans, as locally available or accessible in a nearby facility. Additionally, each hospital had to be staffed with surgical consultants, residents, interns, and medical students. Six out of 17 (35%) facilities met these inclusion criteria, accepted participation, and were randomized.

The participants in this trial were either trauma care frontline personnel or patients with trauma. The eligible trauma care frontline personnel constituted rural trauma team networks and were either surgery residents, intern doctors, third- to fifth-year medical or allied health students, or road traffic police officers concerned with the emergency evacuation of injured patients. It was not permissible for trauma care frontline personnel to offer their services in a way that would cross the treatment arms.

Eligible patient participants were those aged 2‐80 years with motorcycle accident–related neurological and/or musculoskeletal injuries presenting within 24 hours at either the intervention or control hospitals during the study period. The rationale for the restriction of the study to these injuries was threefold. First, to minimize confounding due to diverse injury mechanisms during the impact evaluation of the trauma education intervention. Second, to objectively determine 90-day morbidity using standardized, injury-specific, validated tools such as the Glasgow Outcome Scale for traumatic brain injury (GOS-TBI) and Trauma Outcome Measure Score (TOMS) for musculoskeletal injuries [19,20]. Finally, to attain a sample large enough to reach statistical power in subgroup analyses for the specific injuries.

We excluded medical trainees with no previous exposure to surgical clinical rotation; pregnant women; mentally incapacitated individuals due to psychiatric disorders and without legally authorized representatives to provide informed consent; patients with documented stroke; passengers in a car at the time of collision to control confounding for injury mechanism; and deaths before imaging or arrival at emergency departments.

Prehospital deaths were excluded for 3 reasons. First, the health care system design directs such patients to city mortuaries rather than emergency departments, making accurate assessment of the primary outcome impossible, as our case definition of prehospital time was from the scene to the emergency department. Second, sociocultural norms in rural Uganda, where traditional medicine is prevalent, often discourage postmortems for deaths that are not linked to a public health concern, including postmortem CT imaging, which would hinder analysis of secondary outcomes on all-cause mortality, as caregivers typically view this process as lacking value for money. Finally, the absence of structured “on-scene” emergency care in Uganda at the time of the study meant that recording vital signs before hospital arrival could not be guaranteed, as most patients arrived at emergency departments by public transport; yet, vital signs were essential for assessing injury severity and controlling for potential confounding factors. More detailed eligibility criteria are documented in the trial protocol [21].

### Patient and Public Involvement

We assessed the feasibility of trial outcome tools through engagement with trauma care frontline personnel and patient caregivers before study commencement. Further engagements were conducted by establishing rural trauma teams rooted at the subcounty level and obtaining bidirectional feedback on trauma team performance through weekly audit meetings, as detailed in the study protocols [18,21].

### Sample Size Determination and Power Analysis

The published trial protocol details the power calculations and methodology [21]. The total sample size of 1003 patient participants (501 intervention and 502 control) was estimated using an R shiny application (RStudio, Inc), accounting for the clustering design effect, 80% power, 5% type I error, intracluster correlation coefficient (ICC) of 0.02 (0.01‐0.05), cluster autocorrelation coefficient of 0.8, equal cluster size allowing a coefficient of variation of 0.5, and 18% loss to follow-up [22]. The expected mean difference in primary outcomes was 1.02 hours based on an observational study in the United States [23]. Interim analyses were performed by a blinded statistician on August 28, 2022, in which results showed no patient-reported adverse incidents directly attributable to the intervention, which was the preset termination criteria based on harms; thus, the study continued to completion.

### Data Collection

Data on the specified variables and outcome measures were collected from a piloted MOTOR created for this study. The data were entered into a secure Research Electronic Data Capture (REDCap; Vanderbilt University Medical Center), hosted by the University of Turku in Finland. Qualified medical doctors with 2 years of clinical experience or senior surgery residents served as blinded outcome assessors and were trained to use the MOTOR registry datasheet and *International Classification of Diseases, 10th Revision (ICD-10*) coding system. These assessors integrated data from clinical observations, patient interviews, and hospital and police records. Follow-up included accident and emergency departments, surgical wards, outpatient clinics, and home visits, as outlined in the study protocol [21].

### Study Variables

We captured asymptomatic and symptomatic traumatic brain injury (TBI) based on the National Institute of Neurological Disorders and Stroke case definition. We obtained head CT images for participants who either met the New Orleans criteria or the Canadian CT Head Rule based on local applicable algorithms used at the study sites, whereas we used radiographs to evaluate limb fractures [24]. Based on our baseline study, we obtained variables earlier identified to influence injury outcomes in our settings, including sex, age, comorbidities, injury mechanism, road user category, helmet use, prehospital factors (transportation, first aid, and time), referral decision intervals, head CT results, definitive surgical treatment, multiplicity of injuries, and injury severity based on Glasgow Coma Scale (GCS) and Kampala Trauma Score II (KTS II) [25]. These variables align with the global surgery agenda to improve national surgical care plans in support of universal health coverage and Sustainable Development Goal 3.

### Outcome Measures and Methods of Assessment

Detailed outcome measurements, analysis metrics, and assessment time points are illustrated in the published protocol [21]. The prespecified primary outcomes used measures of central tendency to compare between arms (1) prehospital time (from the accident scene to arrival at the emergency department), and (2) referral dispatch interval (from the time a referral decision was made to the time a patient exited the hospital gate to be transferred to a more resourced facility), measured in hours as indicators of process improvement.

The secondary outcomes compared between arms (1) proportions of all-cause 90-day mortality from the time of injury as a measure of physician-centered clinical outcomes, and (2) morbidity of neurological and musculoskeletal injuries at 90 days from the time of admission using the GOS-TBI (hereafter referred to as Glasgow Outcome Scale [GOS]) and TOMS, respectively, as measures of patient-reported functional outcomes [19,20]. Both GOS and TOMS were measured as final values as reported to outcome assessors, although their baseline equivalents, that is, GCS and Trauma Expectation Factor Score (TEFS), were captured to compare morbidity at baseline [19,26]. GOS was stratified into unfavorable (scores 1‐3) vs. favorable outcomes (scores 4‐5), whereas TOMS was dichotomized into unfavorable (TOMS < TEFS) vs favorable (TOMS ≥ TEFS) in accordance with previous studies [20,27].

These outcome measures were selected to capture the complex, multidimensional effects of trauma from diverse perspectives, including physical, social, and psychological domains. Both the GCS and the GOS have consistently demonstrated excellent internal validity, reproducibility, and reliability in multicenter trials; moreover, the tools have been validated for use in LMICs, with versions applicable in both adult and pediatric populations [20]. These tools can be administered physically, telephonically, or by post and are sensitive to severity and changes in injury sequelae over time in a diverse population of patients and examiners [28].

Acknowledging the importance of end-user involvement in research, the study used patient-reported outcomes (10-item Likert TOMS scale reported as a percentage) to assess the level of pain, physical function, disability, satisfaction with treatment, and overall life satisfaction at 90 days, compared to baseline expectations at admission (10-item Likert TEFS scale). Negative domains such as pain, disability, and activity reduction were assigned a minus score, while positive domains relating to satisfaction with life post-injury received a positive score, with total scores ranging from −700 to 300. Since injuries are not mutually exclusive in clinical settings, both GCS and GOS, or TEFS and TOMS, were administered for patients with both neurological and musculoskeletal trauma who were able to verbalize. These tools have demonstrated excellent psychometric properties in previous studies, as illustrated in Multimedia Appendix 1. KTS II captured the number of serious injuries to control for confounding due to polytrauma. The KTS II was used for its validity and suitability in predicting mortality in LMICs compared to other trauma scores, in addition to its ability to integrate physiological and anatomical indicators such as age-related comorbidities and multiplicity of injuries [29].

### Clinically Meaningful Differences

For prehospital and referral-dispatch intervals, the minimal clinically meaningful difference was established as 1 hour, reflecting the “golden hour” guideline, which stipulates that patients with trauma should reach definitive care within this timeframe to avoid increased mortality [30]. For the GOS-TBI, a 1-point difference was identified as the minimum clinically important difference, as it can shift an individual from an unfavorable to a favorable outcome category and correlates with a 1-point decline in the Disability Rating Scale [31]. This is deemed sufficient to demonstrate meaningful efficacy for TBI care and clinical research interventions, as outlined by the US Food and Drug Administration [31]. Conversely, for the TEFS and TOMS, assessed in the context of rehabilitation and improvements in functional performance from the patients’ perspectives [32], the minimal clinically important difference was defined as the total TOMS score at 90 days meeting or exceeding the patients’ TEFS assessed at admission, consistent with previous studies [33].

### Statistical Analysis

Analyses were performed for all randomized clusters and participants in both intervention and control groups. We tested the normality of the distribution and equality of variance using the Shapiro-Wilk and Levene tests, respectively. For primary outcomes, a 2-sample Wilcoxon rank-sum test was used to compare the median (IQR) due to the skewed nature of the data despite log transformation. For secondary outcomes, we used an adjusted chi-square test to compare the difference in proportions of all-cause 90-day mortality and proportions of unfavorable GOS or TOMS for neurological and/or musculoskeletal injuries, respectively. The median (IQR) time to event (injury to death) was compared using a 2-sample Wilcoxon rank-sum test and visualized using Kaplan-Meier survival estimates. To explicitly address cluster dependency and to overcome SE bias due to the small number of clusters, we compared primary and secondary outcomes between treatment arms and reported cluster-level summaries and effect sizes within the framework of restricted (residual) maximum likelihood (REML) mixed-effects logistic regression models, using the Satterthwaite method of adjustment for the degrees of freedom. We used the Hussey and Hughes stratum-by-time extension in mixed-effects models to examine the variation in outcomes in both treatment arms across multiple time periods to address residual misspecification, assuming no study interruptions, and validated these results using cluster robust SEs (variance-covariance matrix of the estimators) in random-effects models. ICCs within periods were computed using the “.estat icc” command for each outcome using the mixed-effects REML regression models with Satterthwaite adjustment as the ratio of between-cluster variance of outcome to total variance of outcome between and within cluster.

#### Preplanned Subgroup Analyses

We performed preplanned subgroup analyses for sex, road user category, injury mechanisms, severity, and multiplicity of injuries. We used the REML mixed-effects logistic regression models, which permitted adjustment for confounding fixed and random effects to examine factors associated with delayed prehospital and referral-dispatch intervals, all-cause 90-day mortality, and morbidity of neurological and/or musculoskeletal injuries. We used the REML mixed-effects models to accommodate variations in baseline characteristics of random variables, intervention effects, and unbalanced data patterns that could have occurred between treatment groups, resulting from loss to follow-up. The random-effects variable was the treatment arm (intervention vs control) as the unit of analysis, with the odds ratios (ORs) and their corresponding 95% CIs as a direct estimate of the effect size. We examined all covariates for confounding and effect modification using the Cochran-Mantel-Haenszel statistics. Confounders were then added to the REML models individually or in blocks, and percentage change in exposure coefficients, Akaike Information Criterion, Bayesian Information Criterion, and log-likelihood for predictive quality were evaluated. Variables with substantial change in exposure effect were retained, and the models with the smallest Akaike Information Criterion and Bayesian Information Criterion were considered best-fitting. All analyses and data visualization were performed in Stata (version 15.0; StataCorp LLC). We considered *P*<.05 as statistically significant. Due to paper length constraints, ancillary studies detailing results of mixed-effects regression analyses for factors associated with mortality and morbidity of neurological and musculoskeletal injuries, barriers to injury care, and the impact of rural trauma team training on providers’ knowledge, which constituted the tertiary outcomes of this trial, are reported separately [14,34,35] (preprint).

#### Additional Analysis

To account for potential confounding due to factors related to the COVID-19 pandemic’s socioeconomic impact on emergency trauma services and livelihood, trial investigators conducted exploratory nonprespecified analyses comparing outcomes between participants recruited before and after the pandemic in accordance with the CONSERVE guidelines [16]. These analyses were performed to reflect a study halt, restricted participant access during lockdowns, and newly mandated participant safety requirements. Post hoc power analyses were conducted to assess whether the sample sizes used in the study were adequate for detecting effect sizes associated with the various study outcomes, given predetermined assumptions.

#### Handling Missing Data

We compared the baseline characteristics of participants who had missing endpoints due to loss to follow-up between the study arms. We conducted sensitivity analyses to assess the robustness of our findings under different assumptions about missing data. First, we performed a complete case analysis, assuming all missing data were completely missing at random. Second, we considered a worst-case scenario where all participants who lost to follow-up in the intervention group experienced the worst (mortality or unfavorable) trauma outcomes, while all those with missing data in the control group had favorable outcomes or survived. Third, we examined a best-case scenario, assuming all participants with missing data in the intervention group survived with favorable outcomes, while those in the control group had unfavorable outcomes or died. Finally, we performed multilevel multiple imputation to account for clustering and mechanisms of missingness and reported how the interpretation of results varied from the complete case analysis.

### Ethical Considerations

The study adhered to ethical guidelines outlined by the Declaration of Helsinki and the 2017 Uganda National Council for Science and Technology guidelines. Ethical approval was obtained from the research and ethics committees of Mbarara University of Science and Technology (ref MUREC 1/7; 05/05/2019) and from the Uganda National Council for Science and Technology (ref SS 5082) before study commencement. Written informed consent was obtained from all participants or their legally authorized representatives before recruitment. The principal investigator facilitated data collection and trauma team coordination with airtime and transport reimbursement of US $7.50 per participant. This reimbursement was approved by the research and ethics committee to enable research-related follow-up of participants outside of their routine clinical appointments. This paper does not contain any personally identifying individual data or images. This study used data from the piloted MOTOR. At the time of protocol submission, the ethics committee did not mandate prior registration for academic, non–industry-funded proof-of-concept projects (policy effective December 31, 2023). Following the evolution of local policy, the investigators pursued registration with the Pan African Clinical Trial Registry (PACTR202308851460352). Due to COVID-19 disruptions, registration was completed on 08/17/2023, after recruitment commenced. The full protocol for this study was published under the International Registered Report Identifier: DERR1-10.2196/55297. The authors confirm that all related trials were included in this registration [21]. The specific impacts of the pandemic on this trial, including suspension, were reported according to CONSERVE guidelines, and subgroup analyses were performed appropriately to control for this confounding [16]. Where applicable, retrospective registration and protocol deviations due to the pandemic are cited in the abstract and paper text; for completeness, a detailed list of all deviations is provided in Multimedia Appendix 2.

## Results

### Overview

The cluster-level characteristics and resources are provided in Table 1.

**Table 1.** Showing cluster-level characteristics and resources (data source: field data).

Treatment arm	Intervention group			Control group		
Cluster ID^a^ and site-level characteristics	H1^b^	H2^c^	H3^d^	H4^e^	H5^f^	H6^g^
Distance from national referral hospital (km)	209	84	198	150	294	300
Number of functional CT^h^ scanners (n)	^i^0	1	1	1	1	1
Number of functional X-ray machines (n)	1	1	1	1	1	1
Number of functional ultrasound scanners (n)	1	1	1	1	1	1
Number of functional laboratories (n)	1	1	1	1	1	1
Number of functional ambulances (n)	1	2	2	1	2	2
Standard bed capacity (n)	109	500	267	240	300	700
Operating bed capacity (n)	175	600	317	500	333	600
ICU or HDU bed capacity (n)	2	10	20	8	10	12
Number of blood banks (n)	1	1	1	1	1	1
Number of oxygen plants (n)	^j^ 0	1	1	1	1	1
Staffing capacity (% occupancy of sanctioned vacancies), n (%)	169/349 (48)	389/1269 (31)	360/1195 (30)	348/1200 (29)	338/1200 (28)	380/800 (47)
Catchment population (millions)	0.5	4.5	3.5	2.0	3.5	4.0
Geographical coverage, number of cities (number of districts)	0 (6)	1 (11)	1 (9)	0 (5)	1 (9)	0 (10)
Trained permanent staff involved in the care of patients with trauma, n (%)	16/20 (80)	56/70 (80)	32/40 (80)	–0 (0)	–0 (0)	–0 (0)
^k^Trained staff including surgery residents, interns, and medical trainees, n (%)	344/500 (68.8)	87/500 (17.4)	69/500 (13.8)	–0 (0)	–0 (0)	–0 (0)
Patient cluster size for the 12 periods, n (%)	189 (18.8)	140 (14.0)	172 (17.1)	166 (16.6)	183 (18.2)	153 (15.3)
Average cluster size per period (n)	16	12	14	14	15	13

a H: cluster (hospital) identification code (ID).

b H1: Kiryandongo Regional Referral Hospital (public).

c H2: Jinja Regional Referral Hospital (public).

d H3: Hoima Regional Referral Hospital (public).

e H4: Mubende Regional Referral Hospital (public).

f H5: Fort Portal Regional Referral Hospital (public).

g H6: Kampala International University Teaching Hospital (private).

h CT: computed tomography.

i Outsources CT images from neighboring missionary hospital.

j Relies on stored oxygen cylinders and oxygen concentrators.

k Proportions commensurate to the average annual number of interns, surgery residents, and medical trainees received at the respective hospitals.

**Figure 1.**
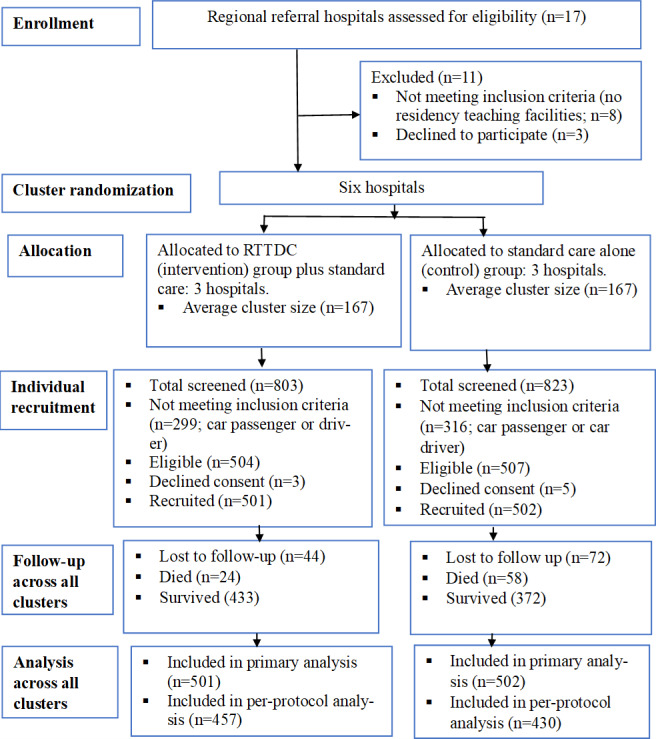
CONSORT (Consolidated Standards of Reporting Trials) flow diagram. RTTDC: Rural Trauma Team Development Course.

At the end of the study period, there were 1003 participants (501 intervention and 502 control), with an average cluster size of 167 in both groups (14 individuals per cluster per period). The participant CONSORT flow diagram is summarized in Figure 1.

### Baseline Sociodemographic and Clinical Characteristics

Cluster- and individual-level baseline sociodemographic and clinical characteristics are summarized in Multimedia Appendix 3. Of 1003 participants, 82% (817) were male and 19% (186) were female (male:female ratio 402:99 in the intervention group vs 415:87 in the control group). The overall median age (IQR) was 28 (22-37) years (median age 28 years, IQR 22-38 years in the intervention group vs median age 28 years, IQR 22-36 years in the control group). There were no significant imbalances in the distribution of baseline characteristics between treatment groups except for 5 out of 37 covariates, namely employment status, proportion sustaining motorcycle-car crash, reported head impact, multiplicity of injuries, and respiratory rate. The overall median injury severity based on KTS II was 8 (IQR 7-9) and did not differ significantly between groups (*P*=·36).

Most participants (77%, 776/1003) arrived by public means (79%, 398/501 in the intervention group vs 75%, 378/502 in the control group). Helmet use was reported at 25% (254/1003; 23%, 144/501 in the intervention vs 28%, 140/502 in the control group). Irrespective of symptoms, 95% (949/1003) of participants reported head trauma (impact). Symptomatic TBI was present in 70% (699/1003; 69%, 347/501 in the intervention vs 70%, 352/502 in the control group). Over 67% (675/1003) of participants met the head CT criteria (67%, 335/501 in the intervention vs 68%, 340/502 in the control group). Conversely, one-third of the participants did not meet the CT criteria. The median GCS was 14 (IQR 11-15) and did not differ between groups (*P*=.12). The admission systolic blood pressure was ≥90 mm Hg in 82% (822/1003), with oxygen saturation >90% in 80% (802/1003) of participants. Loss of consciousness and posttraumatic headache were the most reported clinical presentations. Acute epidural hematomas were the most observed radiological pathology (17%, 175/1003), followed by subdural hematomas (10%, 95/1003). Emergency craniotomy was the most performed neurosurgical intervention (24%, 236/1003). On the other hand, musculoskeletal injuries were present in 80% (799/1003) (80%, 400/501 in intervention vs 80%, 399/502 in control), of which 27% (268/1003) had limb fractures (27%, 134/501 in intervention vs 27%, 134/502 in control).

### Primary Outcome I: Comparison of Prehospital Time

**Figure 2.**
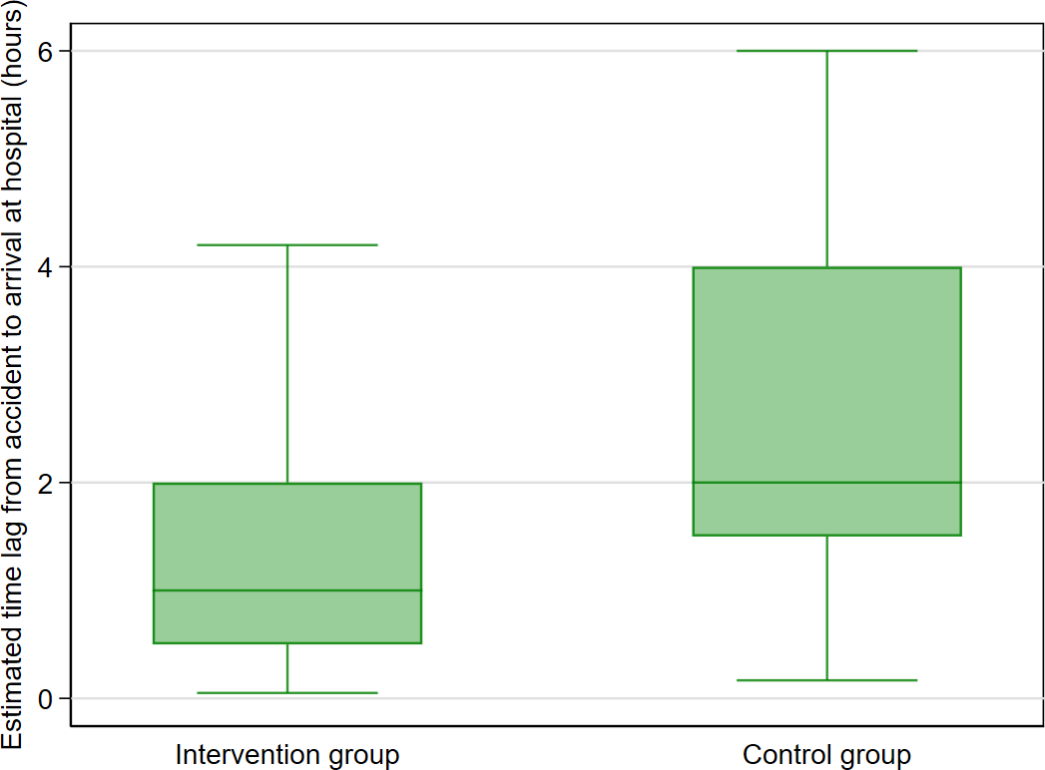
Comparison of median prehospital time lag from accident scenes to arrival at emergency departments.

For the 1003 participants, the overall median prehospital time was 2 (IQR 1.00‐3.00) hours and was shorter in the intervention compared to the control group (1 hour, IQR 0.50‐2.00 hours vs 2 hours, IQR 1.50‐4.00 hours; *P*<.001) (Figure 2). Adjusted predictions of prehospital time between arms across study periods are provided in Multimedia Appendix 4. Factors that contributed to delayed prehospital arrival beyond the optimal 1 hour were commute distances longer than 5 km, being a survivor of motorcycle-static object collisions, needing first aid before arrival at emergency departments, and requiring craniotomy, whereas patients’ age and head CT findings were the most significant determinants of delayed referral-dispatch execution (Multimedia Appendix 5).

### Primary Outcome II: Comparison of Referral Decisions to Dispatch Interval During Interfacility Transfers

**Figure 3.**
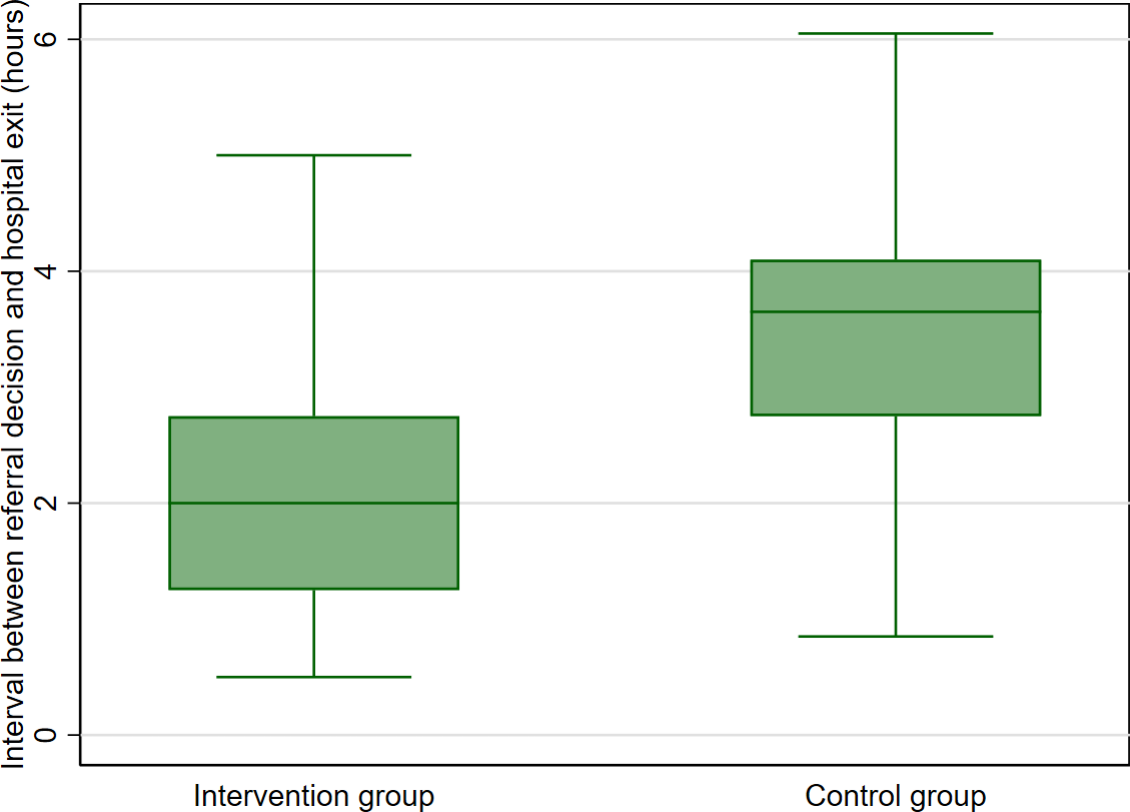
Comparison of referral decision to dispatch interval between groups.

For 69% (691/1003) of patients whose care demands exceeded local resources and warranted referral, the overall median referral decision to dispatch interval was 3 hours (IQR 1.75‐3.85 hours). As provided in Figure 3, the referral decision to dispatch interval was shorter in the intervention (2 hours, IQR 1.25‐2.75 hours) vs the control group (4 hours, IQR 2.50‐4.10 hours; *P*<.001]. Adjusted stratum-by-time predictions of referral-dispatch intervals between arms across study periods are provided in Multimedia Appendix 6.

### Secondary Outcome I: All-Cause 90-Day Mortality

**Figure 4.**
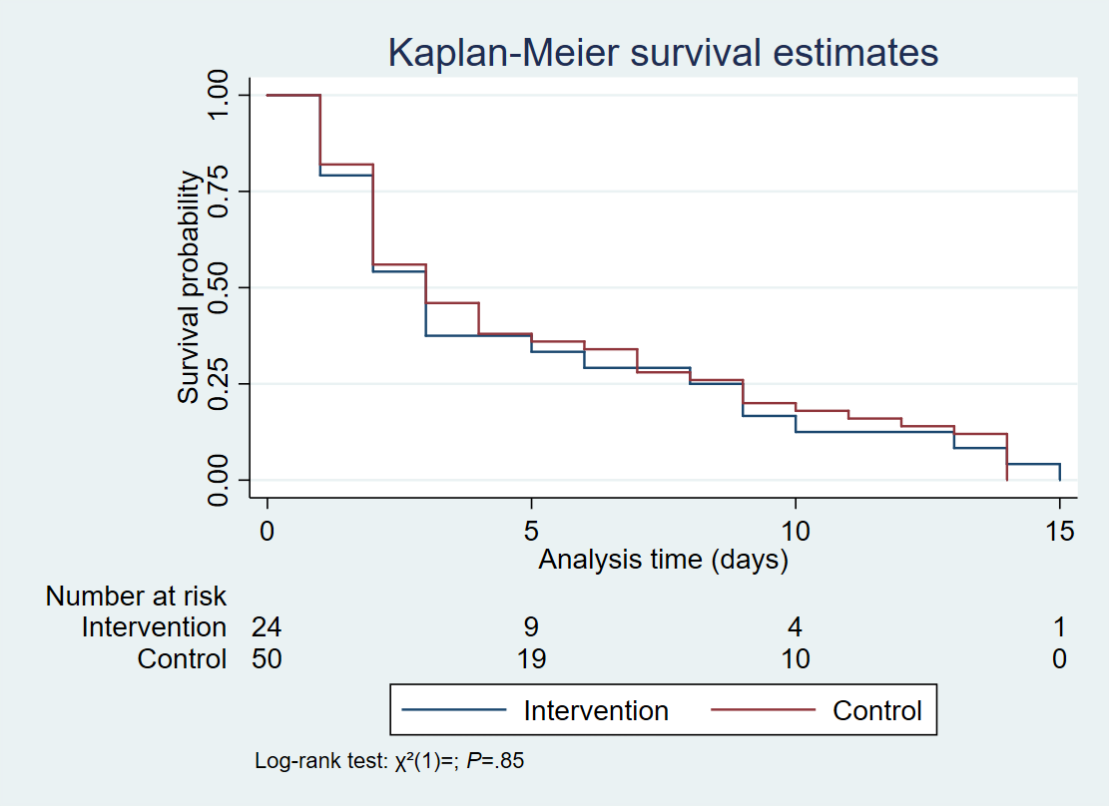
Comparison of Kaplan-Meier survival time (days) between groups.

Of the 88% (887/1003) participants who completed the 90-day follow-up, the overall 90-day mortality was 9% (82/887). Mortality was lower in the intervention at 5% (24/457) compared to the control group at 13% (58/430) (*χ²*_1_=17·9; *P*<.001). Of the 82 deaths, 8 (9.8%) occurred in the emergency departments of the control group vs 0 (0%) in the intervention group. Figure 4 demonstrates that the overall median survival time among the remaining 74 deaths was 3 (IQR 1-8) days and did not differ significantly between groups (*P*=.48). Stratified Cox regression analysis showed that the only determinant of time to event (death) was receiving a craniotomy (*P*=.01), but the proportions that required this procedure did not differ between groups (*P*=.17) (Multimedia Appendix 7). Although more males (84%, 69/82) died than females (16%, 13/82), preplanned subgroup analyses showed that the median survival time did not differ significantly across study periods by sex (*P*=.71) (Multimedia Appendix 8) and by road user category (*P*=.46) (Multimedia Appendix 9).

### Secondary Outcome II: Comparison of Morbidity of Neurological Injuries

Of the 887 out of 1003 participants who completed the 90-day follow-up, 86% (758/887) had a favorable GOS, with a median of 5 (IQR 4-5). The proportion of participants with favorable outcomes was significantly higher in the intervention group (91%, 415/457) compared to the control group (80%, 343/430) (*χ²*_1_=21·7; *P*<.001). The median GOS (IQR) was 5 (4-5) in both arms, with the intervention group showing a slightly higher median compared to the control group (*P*=.003). The results of all GOS subcategories among participants are summarized in Multimedia Appendix 10.

### Secondary Outcome III: Comparison of Morbidity of Musculoskeletal Injuries

**Figure 5.**
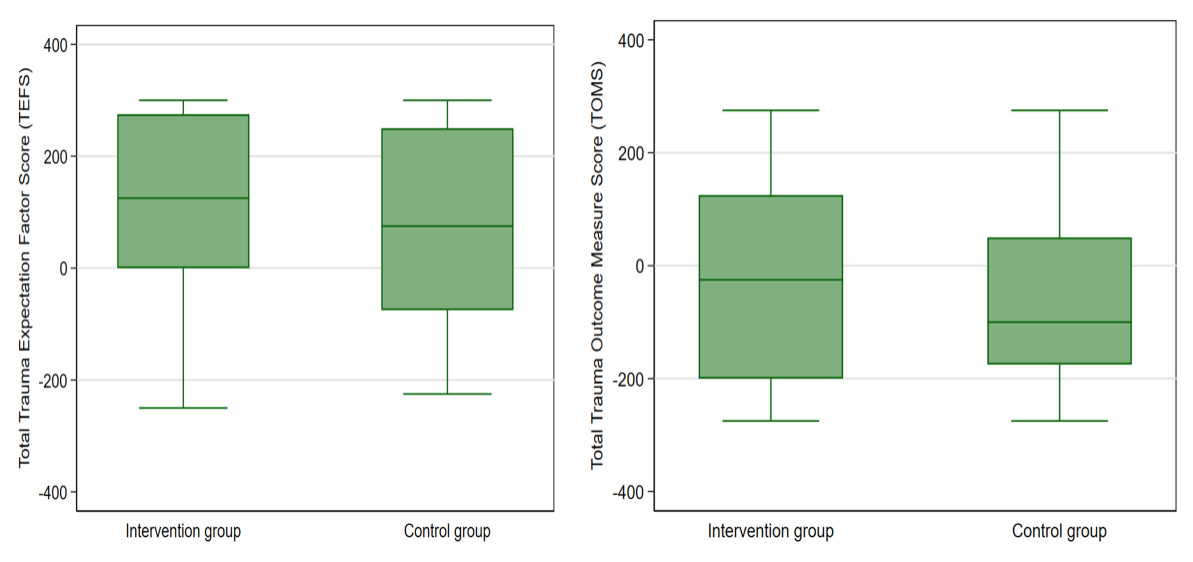
Comparison of Trauma Expectation Factor Scores (TEFSs) and Trauma Outcome Measure Scores (TOMSs) between groups.

Of the 637 out of 799 patients with musculoskeletal injuries whose TEFS and TOMS were complete at 90 days, the majority (75%, 478/637) had a favorable outcome (TOMS ≥ TEFS). As provided in Figure 5, the proportion of patients whose trauma outcome equaled or exceeded their initial expectations was comparable between the intervention (76%, 262/345) vs the control group (74%, 216/292) (*χ²*_1_=0.6; *P*=.57).

**Table 2.** Cluster-level summary and comparison of primary and secondary outcomes between arms (per protocol).

Arm^a^ and cluster ID^b^	Cluster size, (n)	Primary outcome 1: prehospital interval in hours, median (IQR)	Primary outcome 2: referral-dispatch interval in hours, median (IQR)	Secondary outcome 1: 90-day mortality, n (%)	Secondary outcome 2: Proportion of unfavorable GOS^c^, n (%)	Secondary outcome 3: proportion of unfavorable TOMS^d^, n (%)
Both arms	1003	2.0 (1.0‐3.0)	2.8 (1.8‐3.9)	82 (9.2)	129 (14.5)	163 (25.6)
Intervention arm	501	1.0 (0.5‐2.0)	2.0 (1.3‐2.2)	24 (5.2)	42 (9.1)	86 (24.8)
H1^e^	189	1.0 (0.8‐2.0)	2.0 (1.5‐3.0)	15 (8.3)	21 (11.6)	28 (20)
H2^f^	140	1.0 (0.5‐2.0)	1.5 (0.9‐2.1)	6 (5.0)	15 (12.6)	27 (27.3)
H3^g^	172	1.2 (0.5‐2.1)	2.0 (1.2‐2.8)	3 (1.9)	6 (3.7)	31 (28.7)
Control arm	502	2.0 (1.5‐4.0)	3.7 (2.8‐4.1)	58 (13.6)	87 (20.5)	77 (26.6)
H4^h^	166	2.0 (1.0‐3.0)	3.8 (2.8‐4.2)	28 (20.0)	41 (29.3)	21 (25.3)
H5^i^	183	2.0 (1.5‐4.0)	3.6 (2.8‐4.1)	21 (13.2)	28 (17.6)	22 (19.3)
H6^j^	153	2.0 (1.5‐4.0)	3.5 (2.5‐4.0)	9 (7.1)	18 (14.3)	34 (36.6)
Difference (intervention vs. control; 2-sample Wilcoxon rank-sum test *P* value)	—	<.001	<.001	<.001	<.001	.57

a Arm: intervention vs control.

b Cluster ID: unique identifier for each cluster (hospital).

c GOS: Glasgow Outcome Scale.

d TOMS: Trauma Outcome Measure Score.

e H1: Kiryandongo Regional Referral Hospital.

f H2: Jinja Regional Referral Hospital.

g H3: Hoima Regional Referral Hospital.

h H4: Mubende Regional Referral Hospital.

i H5: Fort Portal Regional Referral Hospital.

j H6: Kampala International University Teaching Hospital.

The OR of unfavorable to favorable patient-centered trauma outcomes for musculoskeletal injuries did not differ significantly between intervention (OR 0.952, 95% CI 0.804‐1.128; *P*=.57) vs control (OR 1.073, 95% CI 0.907‐1.269; *P*=.57). This finding remained consistent after preplanned subgroup analyses for limb fractures in the intervention (OR 1.009, 95% CI 0.716‐1.368; *P*=.95) vs in the control group (OR 0.990, 95% CI 0.716‐1.368; *P*=.95) and for isolated tibial fractures in the intervention (OR 0.992, 95% CI 0.740‐1.332; *P*=.96) vs in the control group (OR 1.008, 95% CI 0.726‐1.400; *P*=.96) (Multimedia Appendix 11). Adjusted predictions of TEFS and TOMS across study periods are illustrated in Multimedia Appendix 12. The cluster-level summary and comparison of all outcomes using nonparametric Wilcoxon rank-sum tests are provided in Table 2, with the number of clusters in the intervention arm=3; number of clusters in the control arm=3; total number of clusters: 6; number of discrete decay periods=12.

The results of nonparametric tests were congruent with those of mixed-effects regression (REML) comparisons after adjustment for small-sample correction using the Satterthwaite method (Table 3). The corresponding residual estimates are provided in Multimedia Appendix 13.

**Table 3.** Per-protocol comparison of primary and secondary outcomes across study periods after adjustment for small-sample correction.

Outcome variable (number of observations)	Average cluster size per period, n (min-max)	Average sample per period, n (min-max)	SE	Satterthwaite adjusted df	Coefficient (intervention arm; 95% CI)	*P* value	Residual ICC^a^ at level of study period (95% CI)
Primary outcome 1: prehospital interval in hours (n=1003)	14 (13‐15)	83 (80‐88)	0.08	4	–1.13 (–1.29 to –0.97)	<.001	0.02 (0.00-0.07)
Primary outcome 2: referral-dispatch interval in hours (n=691)	10 (7‐11)	58 (42‐68)	0.08	4	–1.39 (–1.56 to –1.22)	<.001	0.01 (0.00-0.08)
Secondary outcome 1: percentage of 90-day mortality (n=1003)	14 (13‐15)	83 (80‐88)	0.06	4	–0.24 (–0.36 to –0.12)	<.001	0.01 (0.00-0.05)
Secondary outcome 2: percentage of unfavorable Glasgow Outcome Scale (GOS; n=887)	12 (11‐14)	73 (66‐84)	0.02	4	–1.11 (–0.16 to –0.07)	<.001	0.01 (0.00-0.07)
Secondary outcome 3: percentage of unfavorable Trauma Outcome Measure Score (TOMS; n=637)	9 (7-11)	53 (42‐64)	0.04	4	–0.023 (–0.09 to 0.05)	.51	0.02 (0.01-0.10)

a ICC: intracluster correlation coefficient.

### Adverse Events

There were no crossovers between arms, but there were 8 deaths at the emergency department in the control arm, which can be considered as an adverse injury outcome. At 90-day disposition among 805 retained survivors, a total of 16 (2%) participants were still hospitalized due to pressure ulcer sequelae to neurological injuries. These ulcers required long-term debridement and plastic reconstruction alongside physiotherapy and rehabilitation, which all took longer than 90 days. However, the proportions of those retained (7/433 in the intervention vs 9/372 in the control group; *χ*^2^_1_=0.6; *P*=.43) and those discharged home (426/433 in the intervention vs 363/372 in the control group, *χ*^2^_1_=0.01; *P*=.94) did not differ significantly.

To examine the extenuating circumstances of COVID-19, we performed exploratory analyses to probe any differences in outcomes, and our results showed no differences in the prehospital interval, referral decision to dispatch interval, and all-cause mortality before and after the COVID-19 pandemic. However, participants who were seen after the onset of the pandemic were twice as likely to report TOMS below their baseline expectations (OR 2·019, 95% CI 1.178‐3.460; *P*=.01), and this association remained significant even after adjusting for confounding due to age, employment status, marital status, injury mechanisms, injury severity, and multiplicity of injuries (adjusted OR 2.095, 95% CI 1.199‐3.659; *P*=.01). A detailed account of the pandemic effects and other factors that influenced mortality and morbidity is provided here [34].

### Missing Data

Our data quality and handling protocols in REDCap have been previously described in the study protocol [21]. On evaluation of lost to follow-up, the primary outcomes with missing end points (prehospital time or referral dispatch interval) were less than 0.1% (1/1003), whereas 12% (116/1003) were lost to follow-up and had missing secondary outcomes (90-day mortality, GOS, and TOMS). The proportion of loss to follow-up was lower in the intervention (9%, 44/116; *P*=.001) vs in the control group (14%, 72/116*; P*=.001), but the baseline sociodemographic and clinical characteristics of those lost to follow-up did not differ significantly between the 2 treatment groups (all *P*>.05; Table 4).

**Table 4.** Comparison of baseline characteristics among those lost to follow-up. Level of statistical significance: *P*<.05.

Characteristic and category	Intervention group (n=44)	Control group (n=72)	Overall (n=116)	*P* value
Age (years), median (IQR)	28.0 (23.0‐40.5)	30.0 (23‐36.5)	28.0 (23.0‐39.0)	.99
Sex, n (%)				.18
Male	37 (84.1)	54 (75.0)	91 (78.4)	
Female	7 (15.9)	18 (25.0)	25 (21.5)	
Marital status, n (%)				.42
Single	17 (38.6)	28 (38.9)	45 (38.8)	
Married	24 (54.5)	41 (56.9)	65 (56.0)	
Divorced	1 (2.3)	3 (4.2)	4 (3.4)	
Other	2 (4.5)	0 (0.0)	2 (1.7)	
Employment, n (%)				.37
Formal paid employment	1 (2.3)	5 (6.9)	6 (5.2)	
Self-employed	68.2 (41.7)	51 (70.8)	81 (69.8)	
Unemployed	2 (4.5)	4 (5.6)	6 (5.2)	
Student	9 (20.5)	12 (16.7)	21 (18.1)	
Other	2 (4.5)	0 (0.0)	2 (1.7)	
Commute distance, median (IQR)	4 (2.0‐17.0)	5 (3.0‐12.5)	5 (2.7‐14.0)	.53
Road user category, n (%)				.06
Passenger	11 (25.0)	32 (44.4)	43 (37.1)	
Pedestrian	9 (20.5)	16 (22.2)	25 (21.6)	
Motorcyclist	24 (24.5)	24 (33.3)	48 (41.4)	
Mode of arrival, n (%)				.90
Ambulance	4 (9.1)	9 (12.5)	13 (11.2)	
Public means (taxi or cycle)	39 (88.6)	61 (84.7)	100 (86.2)	
Other	1 (1.4)	2 (2.8)	3 (2.6)	
Prehospital care (first aid) received?				.71
Yes	26 (59.0)	40 (55.6)	66 (56.9)	
No	18 (40.9)	32 (44.4)	50 (43.1)	
Who gave first aid?				.83
Health worker	14 (31.8)	24 (33.3)	38 (32.8)	
Lay bystander/police	4 (9.1)	8 (11.1)	12 (10.3)	
Mechanism of injury, n (%)				.36
Motorcycle-motorcycle crash	19 (43.2)	41 (56.9)	60 (51.7)	
Motorcycle-pedestrian	13 (29.5)	20 (27.8)	33 (28.4)	
Motorcycle-car crash	9 (20.5)	9 (12.5)	18 (15.5)	
Motorcycle static object	3 (6.8)	2 (2.8)	5 (4.3)	
Comorbidity (chronic medical illness present), n (%)				.40
Hypertension	3 (100.0)	1 (33.3)	4 (66.7)	
Asthma	0 (0.0)	2 (66.7)	2 (33.3)	
Injury severity (KTS)^a^, n (%)				.68
Mild (9-10)	25 (56.8)	41 (56.9)	66 (56.9)	
Moderate (7-8)	14 (31.8)	26 (36.1)	40 (34.5)	
Severe ≤6	5 (11.4)	5 (6.9)	10 (8.6)	
Score for serious injuries, n (%)				.66
>1	4 (9.1)	8 (11.1)	12 (10.3)	
1	32 (72.7)	52 (72.2)	87 (75.00	
None	5 (11.4)	12 (16.7)	17 (14.7)	

a KTS: Kampala Trauma Score.

Given the statistically significant differential attrition in loss to follow-up between arms, we simulated the base, worst-, and best-case scenarios. We found that overall, missing data did not impact the interpretation of results of both primary and secondary outcomes under the different scenarios except for mortality (14%, 68/483 in the intervention vs 12%, 58/470 in the control group; *P*=.34) and morbidity based on unfavorable GOS (1-3) for neurological injuries (14%, 68/483 in the intervention vs 19%, 87/470 in the control group; *P*=.06), which did not reach statistical significance in the worst-case scenario (Multimedia Appendix 14). However, the results from multiple imputations were congruent with complete-case analysis, with all primary and secondary outcomes (mortality and GOS) reaching statistical significance (all *P*<.01; Table 5). Note that the estimates for random effects parameters were computed in mixed effects restricted maximum likelihood (REML) regression models using the Satterthwaite method of adjustment of degrees of freedom for small sample correction. Number of study arms=2 (intervention vs control); number of discrete time decay study periods=12; total number of clusters=6; number of clusters per treatment arm=3; sample size may vary due to variation in study outcomes. All analysis variables were included in the model regardless of completeness. The summary presented is a pooled analysis of 20 imputations. Imputed is the minimum across “*m*” of the number of filled-in observations. Imputation method: Multiple imputation by chained equation (MICE). Number of imputations (*m*)=20. Assumption: Missing at random or completely missing at random. . The corresponding imputed variables with loss to follow-up (missing data) are provided in Multimedia Appendix 15, whereas the residual estimates after multiple imputation are provided in Multimedia Appendix 16.

**Table 5.** Multiple imputation and comparison of primary and secondary outcomes across study periods after adjustment for small sample correction.

Outcome variable (no of observations)	Average cluster size per period, n (min-max)	Average sample per period, n (min-max)	SE	Satterthwaite adjusted df	Coefficient (intervention group; 95% CI)	*P* value	Residual ICC^a^ at level of study period [95% CI]
Primary outcome 1: prehospital interval (hour; n=1003)	14 (13‐15)	83 (80‐88)	0.08	4	–1.14 (–1.30 to –0.98)	<.001	0.02 (0.00-0.07)
Primary outcome 2: referral-dispatch interval (hour; n=691)	10 (7‐11)	58 (42‐68)	0.08	4	–1.41 (–1.57 to –1.25)	<.001	0.01 (0.00-0.08)
Secondary outcome 1: percentage of 90-day mortality (%; n=1003)	14 (13‐15)	83 (80‐88)	0.06	4	–0.24 (–0.35 to 0.11)	<.001	0.01 (0.00-0.06)
Secondary outcome 2: percentage of unfavorable Glasgow Outcome Scale (GOS^b^; %; n=1003)	14 (13-15)	83 (80‐88)	0.02	4	–1.10 (–0.14 to 0.06)	<.001	0.01 (0.00-0.07)
Secondary outcome 3: percentage of unfavorable Trauma Outcome Measure Score (TOMS^c^; %; n=637)	9 (7-11)	53 (42‐64)	0.04	4	–0.02 (–0.09 to 0.05)	.56	0.03 (0.01-0.10)

a ICC: intracluster correlation coefficient.

### Posthoc Power Analyses

The posthoc analyses indicated that, except for secondary outcomes related to the TOMS for musculoskeletal injuries, the sample sizes used in the study were sufficient for detecting effect sizes associated with the various study outcomes at a power level of 80%, based on both predetermined and observed ICC and study assumptions (Multimedia Appendix 17). We did not apply corrections for multiple testing, as the study used marginal rather than disjunctive power. However, with our 5 key study outcomes at a significance level of *P*<.05, the differences in primary outcomes (prehospital time and referral-dispatch interval) would remain statistically significant at *P*<.01 if the Bonferroni method (ie, 0.05/5) were used. Furthermore, in our exploratory analyses of secondary outcomes with distinct constructs (ie, mortality, GOS, and TOMS), the risk of missing a potentially significant finding was deemed a greater concern than the occurrence of false positives.

## Discussion

### Principal Findings

To our knowledge, this study represents a pioneering cluster-randomized trial assessing the impact of a medical education program (RTTDC) on clinical processes and patient outcomes in an LMIC setting. This trial found that RTTDC training significantly reduced prehospital time, highlighting its potential for improved budget allocation for prehospital logistical systems aimed at enhancing care for neurological and orthopedic injuries in these settings. Prior research, primarily observational or quasi-experimental, has indicated similar reductions in referral decision times and transfer intervals; for instance, a 1.91-hour decrease was reported in Western Virginia hospitals compared to control groups [36]. Further findings from this trial indicated a reduction in the interval from referral decision to hospital dispatch for patients who required interfacility transfer (*P*<·001). These results align with a recent study involving 472 trauma patients in the United States, which documented a similar decrease in emergency department dwell time [7]. However, these studies often faced limitations due to small sample sizes and potential biases. Poor interhospital coordination, dysfunctional referral systems, and transportation were the most important contributing factors to delayed emergency surgical access in rural settings, alongside human, financial, and infrastructural resource challenges, as this trial demonstrated that most trauma patients arrived by public means, which may apply to other LMICs.

The trial’s overall 90-day mortality rate was comparable to prior Ugandan studies but higher than rates reported in the US and European cohorts [37,38]. Notably, the intervention group exhibited significantly lower mortality rates than the control group in both complete-case analysis and best-case scenarios, suggesting that RTTDC implementation and the coordination of rural trauma teams may effectively minimize mortality associated with prolonged referral decision times. In this trial, referral decision to dispatch time of more than 1 hour was associated with a fourfold increase in mortality after controlling for confounding due to injury mechanisms and severity. This finding is particularly relevant given that delays in care have been shown to exacerbate injury-related mortality in both LMICs and high-income countries [37]. Consistent outcomes were observed in previous studies, including a notable 50% reduction in mortality rates associated with RTTDC training in the United States [23]. Despite these potential positive results, variations in mortality outcome rates across studies exist and may stem from different methodological approaches, sample sizes, and settings [7]. In this study, the mortality rate and operative neurosurgical interventions were relatively high, partly due to the restriction of the study to motorcyclists in rural settings where helmet usage and speed limits are poorly regulated, resulting in severe TBI. In the case of 8 deaths in emergency departments of control centers, it was determined that patients were awaiting head CT scans at the request of trainee general surgery residents to decide on transfers for neurosurgical evaluation. However, no local neurosurgeons were available to address potential surgical needs based on the CT results. This highlights the lesson from RTTDC that investigations should not be requested without the requisite human resources to act on the findings.

In our settings, the impact of RTTDC was driven by the actualization of physical trauma teams through pairing junior and senior medical trainees, effective communication and coordination at the community level, facilitated by trained volunteer traffic police, and guided by interns and surgery residents in emergency departments. This collaboration ensured proper selection of destination hospitals, prearrival preparations, and swift interfacility transfers for injuries exceeding local capacity. Key strengths of RTTDC included emphasizing teamwork, closed-loop communication within trauma teams, and optimizing local resources based on facility capabilities. These are elements that other trauma education courses in LMICs should replicate. Recognizing that forgetting is a barrier to skill application, we reinforced knowledge transfer through weekly clinical audits and regular feedback to trauma teams, which helped maintain focus on course objectives. This decision-making support was critical, as previous research indicated that only 31% of district and regional referral hospitals in Uganda had emergency unit protocols [39].

Additionally, prior trauma education interventions, such as the Kampala Advanced Trauma Course, showed limited impact on facility-wide best practices when specialist surgeons and experienced medical officers were the main participants due to inadequate skill transfer to junior faculty [40]. Our experience in audit meetings revealed that local consultant surgeons, although willing to assist, were sometimes unreachable when surgical residents required their expertise in emergency situations. We found the “train the trainer” approach effective for fostering team cohesion by pairing third-year medical students with fifth-year students, interns, and residents during night duty calls. Therefore, a comprehensive understanding of the strengths and weaknesses of the health care system for which a trauma course or a medical education program is to be implemented is critical for its effectiveness. Our study underscores the importance of involving frontline workers, such as trainee surgeons, in trauma training initiatives. To address this gap, 3 local universities in Uganda have initiated postgraduate medical education programs in emergency medicine, although these roles are not yet integrated into the national civil service staffing structure by the government. Long-term local trauma fellowship programs can accelerate this goal and address issues of sustainability.

Knowledge reinforcement through support audit meetings also emphasized the necessity of routine community engagement with traffic police, as there was no structured approach to enhancing community awareness or providing emergency care at accident scenes in Uganda during the study. Ideal Level II and III trauma centers should implement trauma quality assessment and outreach programs for their communities. The meetings revealed practical insights into team dynamics and technological resource challenges, including network connectivity and difficulties in locating ambulances. These issues were addressed by coordinating with volunteer taxi drivers who facilitated rescue evacuations under the guidance of traffic police, general surgery trainees, or intern physicians. This strategy improved the selection of destination facilities and enabled concise handover reports from police, who are first responders in most emergencies, since only 25% of Uganda’s 48 million population had access to ambulances [13].

These lessons highlight the urgent need for investment in surgical education, emergency medical services (EMSs), telemedicine, and remote monitoring systems such as edge computing to address connectivity issues, including the establishment of toll-free emergency numbers that link police to hospitals and ambulances. Additionally, further medical training for lay responders and efforts to increase the number of paramedics are essential. Uganda’s national EMS policy, launched in 2021, aims to enhance emergency care capacity, but fixed staff occupancy in district hospital emergency departments remains below 50% [39,41]. Integrating artificial intelligence–enabled triage, referral decision support, and imaging prioritization into rural trauma workflows may overcome some of the human resource constraints to reduce delays in the trauma care continuum in low-resource settings. However, enhancing patient outcomes through artificial intelligence in these contexts necessitates robust ethical oversight, fair dataset representation from LMICs, and ongoing validation via prospective clinical trials [42].

Contemporaneous evidence indicates that community responders play a significant role in both current and future EMS, even in countries with established prehospital care systems, such as the United Kingdom, due to their timely responses and reduced maintenance costs [43]. The Ugandan EMS policy acknowledges the potential contributions of community responders [41]. However, to actualize these contributions, it necessitates a separate budget to reimburse costs associated with communication and transportation, support training that bridges lay responders to clinicians, and facilitate medical interns and residents who work overtime to address staffing shortages in emergency departments (resource optimization). Moreover, it is essential to design legal protections for community responders through Good Samaritan laws, establish clear standard operating procedures with decision support algorithms for trainee physicians, develop referral protocols with automated workflow, and implement performance measurement frameworks for improvement. These measures would prevent fatalities during transport, including auditing incidents that occur before ambulance arrival [44]. Consequently, it is crucial for medical training institutions, public-private partnerships, ambulance and insurance companies, and civil society organizations to consider investing in this area.

In addition to mortality, the study explored morbidity outcomes, wherein the intervention group had a lower proportion of unfavorable GOS-TBI scores compared to controls, indicating improved recovery trajectories. However, the analysis of TOMS for musculoskeletal injuries was inconclusive, possibly due to the study being underpowered for this particular outcome, a higher proportion of polytrauma cases, and higher initial trauma outcome expectations among intervention participants. Existing literature corroborates the increased morbidity associated with polytrauma, emphasizing the need for future research into the long-term outcomes of RTTDC interventions in the context of multiple injuries [45]. On the other hand, the nonsignificant results for musculoskeletal injuries may stem from the inherent focus of the RTTDC on life-saving resuscitation measures and the immediate transfer of critical cases such as neurotrauma, which require high care demands. Alternatively, it may be that the evaluation tools (TEFS and TOMS) are less sensitive to assessing outcomes related to musculoskeletal injuries within the trauma education context. To resolve this uncertainty, further investigation is warranted with a larger number of clusters and cluster sizes.

### Policy Implications

The implications of our findings are threefold. First, while RTTDC training shows promise for enhancing clinical processes and patient outcomes in LMICs, local and regional evaluations of its impact are essential for effective implementation. Establishing regional continuing medical education certification centers and long-term postgraduate trauma training programs for trauma care providers could facilitate this goal and address concerns about sustainability. Second, the development of context-specific rural trauma transfer guidelines is critical, incorporating local norms and logistical realities. This may necessitate broader participation from various health care professionals, including medical trainees, private ambulance companies, and lay traffic police who provide immediate crash responses, to strengthen rural trauma systems. Finally, a tailored needs assessment for each LMIC is crucial to adapt trauma education programs, ensuring they address specific cultural, technological, and systemic challenges while promoting sustainability. Existing trauma education programs in LMICs lacked robust impact evaluation, cohesive local contextualization, and common outcome metrics for reporting [3]. This trial provided critical clinical processes and patient-reported indices that could constitute a core outcome set for interventions linking medical education to patient outcomes in a trauma context.

### Study Strengths and Limitations

This trial offers significant strengths in evaluating a locally contextualized trauma education program and linking it to clinical outcomes in resource-limited rural settings. It effectively demonstrates that training trauma care frontline workers, such as voluntary traffic police officers and medical trainees, can enhance clinical practices and patient outcomes. We previously highlighted the impact of RTTDC on care providers’ knowledge and behavioral change [14]. This trial builds on that foundation by showing the translation of acquired classroom knowledge into improved clinical processes. Early engagement with frontline workers ensured that the trial was relevant, and the findings may inform future core outcome sets for similar trials in LMICs, where this study forms an integral component of pioneering efforts to evaluate the real-world impact of an education program (RTTDC) in controlled clinical settings. Studies of this nature are topical for the global surgery community. This study provides an ideal example of how equitable South-West partnerships in global surgery should be implemented. A detailed statement regarding how partnerships and collaborations were actualized is available in Multimedia Appendix 18. There are no previous large-scale cluster randomized controlled trials that have evaluated the effect of trauma education programs on patient workflows and clinical outcomes, but a recent feasibility trial evaluating ATLS course and PTC against standard care in India found justification for full-scale cluster randomized trials [46], thus our publicly available data in the Multimedia Appendix 19 is timely [47].

However, this study has 6 notable limitations. First, a higher loss to follow-up in the control group may introduce potential systematic attrition bias, which must be addressed in future studies by assuming the worst-case situation during sample size computation. Although the overall dropout rate (11.6%) remains within acceptable limits, the conclusions regarding mortality and morbidity must be cautiously interpreted in this context. The differential attrition could suggest that missingness was likely related to the effectiveness of the intervention, with plausibility that participants in the control group without perceived improvement due to unfavorable trauma outcomes or other unmeasured postrandomization experiences were more likely to be lost to follow-up. Furthermore, the ICC was estimated based solely on primary outcomes, which may not accurately reflect variations in cluster sizes for secondary outcomes, as observed with TOMSs for musculoskeletal injuries. This is a common issue in cluster-randomized trial design.

Second, some patients at intervention sites may not have received care from trained providers, limiting the intervention’s individual-level impact. Due to significant shortages of health care personnel in Uganda, we set the trained staff capacity in emergency units at 80%, as complete participation in training was not feasible at any given time. This strategy addressed human resource constraints while minimizing ceiling effects associated with training and fostering information sharing among trauma team members in our train-the-trainer model. Given our trauma “team approach” to injury care, which deployed a 5-member in-hospital team for each patient, it was not feasible to provide cluster-level proportions of trained providers and their association with outcomes. However, 80% coverage may result in occasional individual-level exposure misclassification, with a 1:4 likelihood that a patient may encounter 1 untrained vs 4 trained providers during the continuum of care within a typical 5-member trauma team at the same intervention hospital. Consequently, any potential biases in estimates are likely to trend away from the null.

Third, the generalizability of findings is constrained to those who underwent head CT scans in a single country’s health care system, excluding emerging prognostic factors for neurological injuries such as blood-based biomarkers.

Fourth, although exploratory analyses indicated no significant changes in prehospital time due to the COVID-19 pandemic, its broader social effects on hospital surroundings, resuscitative capability, injury patterns, and patient-reported morbidity outcomes remained challenging to quantify. Moreover, COVID-19’s extenuating effects on the trial, including study halt, retrospective registration, and need for posthoc data stratification by pandemic status, which was not hypothesized beforehand, could introduce bias. However, we are unaware of any other events that could have influenced referral decisions and morbidity outcomes.

Fifth, the absence of preintervention data at the study sites represents a limitation. Ideally, if data had been available before training, a comparison of injury outcomes before and after the training would have been preferable to the current approach of making parallel comparisons. Moreover, the data constraints on prehospital deaths may systemically bias estimates of prehospital time and mortality outcomes, introducing survivor bias. For instance, we could not establish if the intervention reduced prehospital deaths, an issue the Ugandan government can address by mandating all unnatural prehospital deaths to undergo postmortem and forensic analysis to determine the cause of death. Although there were 8 mortalities in the emergency department of control hospitals vs zero in the intervention group, and the principal determinant of time-to-event (death), that is, requiring a craniotomy, did not differ between groups, which would trend any biased estimates away from the null; the authors recommend that the study conclusions should only be applied to patients surviving up to hospital arrival.

Finally, despite sensitivity analyses, we are aware that the relatively smaller number of clusters and cluster sizes, and aggregating intracranial lesions as axial or extra-axial or dichotomizing the GOS and TOMS tools as favorable vs unfavorable outcomes, could have led to loss of information and left unmeasured residual confounding that can potentially contribute to some of the observed associations. Moreover, we were also unable to account for the heterogeneity across the considerably wide range of our participants’ age groups, despite trauma not being segregative, an issue future studies should address.

### Conclusions

This trial provided reasonable evidence to suggest that rural trauma team development training potentially decreased prehospital time and referral decision intervals, lowering motorcycle accident–related mortality from neurological and musculoskeletal injuries, without negatively impacting patient-reported morbidity. Despite potential measurement biases from the exclusion of prehospital deaths due to data constraints and the relatively smaller number of clusters, these findings highlight the importance of enhancing rural trauma team capabilities and supporting ongoing investment in global trauma education programs. Future research should focus on large, multicountry studies using long-term trauma registries with diverse injury mechanisms and patient-centered quality-of-life outcome measures tailored to local contexts in LMICs.

## Supplementary material

10.2196/82591 Multimedia Appendix 1 Psychometric properties of data collection tools and interpretation.

10.2196/82591 Multimedia Appendix 2 Protocol deviations and implementation fidelity.

10.2196/82591 Multimedia Appendix 3 Baseline sociodemographic and clinical characteristics of participants.

10.2196/82591 Multimedia Appendix 4 Adjusted predictions of prehospital time between arms across study periods, with CIs.

10.2196/82591 Multimedia Appendix 5 Factors associated with delayed prehospital time and referral-dispatch interval of more than 1 hour.

10.2196/82591 Multimedia Appendix 6 Adjusted stratum-by-time predictions of referral-dispatch interval between arms across study periods.

10.2196/82591 Multimedia Appendix 7 Stratified Cox regression analysis of determinants of time to event (death).

10.2196/82591 Multimedia Appendix 8 Subgroup analysis of survival time (days) stratified by sex.

10.2196/82591 Multimedia Appendix 9 Subgroup analysis of survival time (days) by road user category.

10.2196/82591 Multimedia Appendix 10 Comparison of Glasgow Outcome Score at 90 days between treatment groups.

10.2196/82591 Multimedia Appendix 11 Subgroup analysis of Trauma Expectation Factor Score (TEFS) and Trauma Outcome Measure Score (TOMS) for participants with tibial fractures.

10.2196/82591 Multimedia Appendix 12 Trauma Expectation Factor Scores (TEFSs) and Trauma Outcome Measure Scores (TOMSs) across study periods.

10.2196/82591 Multimedia Appendix 13 Corresponding residual estimates.

10.2196/82591 Multimedia Appendix 14 Sensitivity analyses of injury outcomes under different case scenarios.

10.2196/82591 Multimedia Appendix 15 Imputed variables with loss to follow-up missing data.

10.2196/82591 Multimedia Appendix 16 Residual estimates for random effects parameters after multiple imputations for missing data.

10.2196/82591 Multimedia Appendix 17 Post hoc power and sample size analyses for various observed study outcomes.

10.2196/82591 Multimedia Appendix 18 Reflexivity statement regarding collaborative partnerships.

10.2196/82591 Multimedia Appendix 19 Data analysis code and dataset.

10.2196/82591 Checklist 1 CONSORT (Consolidated Standards of Reporting Trials) 2025 checklist.

10.2196/82591 Checklist 2 CONSORT (Consolidated Standards of Reporting Trials) 2010 guidelines for cluster randomized trials.

10.2196/82591 Checklist 3 Consolidated Standards of Reporting Trials and SPIRIT Extension for Randomized Controlled Trials Revised in Extenuating Circumstances (CONSORT-CONSERVE) checklist version 2021.

## Abbreviations

ATLS: Advanced Trauma Life Support
CONSERVE: CONSORT and SPIRIT Extension for Randomized Controlled Trials Revised in Extenuating Circumstances
CT: Computed Tomography
EMS: Emergency Medical Service
GCS: Glasgow Coma Scale
GOS: Glasgow Outcome Scale
GOS-TBI: Glasgow Outcome Scale for Traumatic Brain Injury
ICC: Intracluster Correlation Coefficient
*ICD-10*: *International Classification of Diseases, 10th Revision*
KTS II: Kampala Trauma Score II
LMIC: Low- and Middle-Income Country
MOTOR: Motorcycle Trauma Outcome Registry
OR: Odds Ratio
PTC: Primary Trauma Course
REDCap: Research Electronic Data Capture
REML: Restricted Maximum Likelihood
RTTDC: Rural Trauma Team Development Course
TBI: Traumatic Brain Injury
TEFS: Trauma Expectation Factor Score
TOMS: Trauma Outcome Measure Score
