# Supplementary material for: Impact of Rural Trauma Team Development Education on Prehospital Time, Referral-to-Dispatch Interval, and Neurological and Musculoskeletal Injury Outcomes: Cluster Randomized Controlled Trial
Source: JMIR Hum Factors. 2026 Apr 20;13:e82591. doi: 10.2196/82591 (PMC13094805; doi:10.2196/82591)
Supplement: Multimedia Appendix 1 [file humanfactors-v13-e82591-s001.docx]

Multimedia Appendix 1: Psychometric properties of data collection tools and interpretation.

We used the Glasgow Coma Scale (GCS) to evaluate the severity of traumatic brain injury (TBI) in acute phase based on eye opening, verbal, and motor response; each with a minimum score of one and maximum score of 4, 5 and 6 respectively; totaling to (3-15) [1]. The Glasgow Outcome Scale (GOS-TBI) is a global outcome scale that was used to ascertain functional independence, putting into context the mental and cognitive aspects of life after injury [2], [3]. The GOS was assessed on a five-point scale as (1=death, 2=persistent vegetative state, 3=severe disability, 4=moderate disability, 5=good recovery) which overcomes the poor inter-rater reliability for borderline subcategories such as upper and lower disability in the extended version of the scale (GOS-E), when assessed by multiple assessors in multicenter studies [2].

On the other hand, the Trauma Expectation Factor Score (TEFS) and Trauma Outcome Measure Score (TOMS) [4], were used to evaluate patient-centered morbidity outcomes for musculoskeletal injuries. TEFS/TOMS were used to determine the extent of (a) pain, (b) physical function, (c) disability, (d) satisfaction with injury treatment, physical appearance, and overall satisfaction with life after injury, in comparison to the initial trauma expectations at baseline.

For instance, in the first week of admission, based on their perceived pre-injury state, the 10-item Likert TEFS asked participants of their expectations at 90 days post-injury treatment as follows:

Domain (a) pain: on scale of zero to 100% (0=no pain, 25%=mild pain, 50%=moderate pain, 75%=severe pain, 100%=very severe unbearable pain), (i) how painful do you expect your injury to be 12 weeks after treatment?

Domain (b) physical function: on a scale of zero to100% (0=no interference, 25%=mild interference, 50%=moderate interference, 75%=severe interference, 100%=very severe unbearable interference); how much do you anticipate your injury to interfere with your usual: (ii) daily activity (walking, standing, climbing stairs, driving , sleeping)? (iii) work (housework, school, recreation/sports)? (iv) activities of daily living (eating, dressing, wearing shoes)? (v) relationships (family, friends, coworkers)?

Domain (c) disability and activity limitation: on a scale of zero to 100% (0% reduction, 25% reduction 50% reduction%, 75% reduction, 100% reduction); how much do you expect to cut down on: (vi) physical activities that are *necessary* to do including work, housework, and school? (vii) optional physical activity *you enjoy doing* including sports, recreation, gardening?

Domain (d) satisfaction with life after injury: on a scale of zero to 100% (0% satisfied, 25% satisfied, 50% satisfied, 75% satisfied, 100% satisfied); how satisfied do you expect to be with your: (viii) level of pain, physical function, and disability? (ix) physical appearance due to injuries? (x) overall wellbeing?

The 10-item Likert scale of TOMS asked similar questions at 90 days regarding the status of each domain item e.g., on scale of zero to 100% (i) how painful in your injury today? How much does your injury currently interfere with your usual (i) necessary activity? (iii) physical activity? (iv) activities of daily living? (v) relationships? How much do you currently cut down on your: (vi) necessary physical activity, (vii) physical activities you enjoy doing? And to what extent are you currently satisfied with your: (viii) level of pain, physical function, and disability? (ix) physical appearance of your injuries? (x) overall wellbeing?

To determine which domains dominated and if patients met their trauma outcome expectations within the context of their perceived preinjury status, the seven domain items with negative impacts on life such as (a) pain, (b) interference with physical function, and (c) activity cutdown due to disability limitations were assigned a minus score whereas the three domain items relating to satisfaction with life after injury were assigned a positive score; thus the total score for all 10-tems in either TEFS or TOMS ranged from (-700 to 300) as detailed the data collection tool [5]. Since neurological and musculoskeletal injuries are not mutually exclusive in clinical settings, both GCS/GOS and TOM/TETS were administered for those with multiple injuries who were able to verbalize, and the number of serious injuries were captured in Kampala Trauma Score (KTS) to control for confounding due to poly trauma. These data collection tools have demonstrated good to excellent psychometric properties in previous studies as summarized in (Table S1) below.

**Table S1:** Psychometric properties of data collection tools.

| Tool | Internal consistence (Cronbach Alpha) | Reliability (Intraclass Correlation Coefficient) | Reproducibility  (Weighted Kappa) | Responsiveness to patients’ condition over time (Wald Test) | Study |
| --- | --- | --- | --- | --- | --- |
| TEFS | >0.87 | >0.90 | ≥0.67 | P<0.001 | [4], [6] |
| TOMS | >0.87 | >0.90 | ≥0.92 | P<0.001 | [4], [6] |
| GOS | 0.85 | 0.89 | 0.92 | P<.001 | [3], [7] |
| GCS | ≥0.80 | ≥0.85 | ≥0.75 | - | [8] |
| KTS | 0.70-0.85 | 0.80-0.95 | ≥0.80 | - | [9], [10] |

References:

[1] S. Jain and L. M. Iverson, “Glasgow Coma Scale.,” Treasure Island (FL), 2024.

[2] L. Wilson *et al.*, “A Manual for the Glasgow Outcome Scale-Extended Interview.,” *J. Neurotrauma*, vol. 38, no. 17, pp. 2435–2446, Sep. 2021, doi: 10.1089/neu.2020.7527.

[3] T. McMillan, L. Wilson, J. Ponsford, H. Levin, G. Teasdale, and M. Bond, “The Glasgow Outcome Scale — 40 years of application and refinement,” *Nat. Rev. Neurol.*, vol. 12, no. 8, pp. 477–485, 2016, doi: 10.1038/nrneurol.2016.89.

[4] M. Suk *et al.*, “TEFTOM: A Promising General Trauma Expectation/Outcome Measure-Results of a Validation Study on Pan-American Ankle and Distal Tibia Trauma Patients,” *ISRN Orthop*, vol. 2013, p. 801784, 2013, doi: 10.1155/2013/801784.

[5] H. Lule *et al.*, “Effect of Rural Trauma Team Development on the Outcomes of Motorcycle Accident–Related Injuries (Motor Registry Project): Protocol for a Multicenter Cluster Randomized Controlled Trial,” *JMIR Res. Protoc.*, vol. 13, p. e55297, May 2024, doi: 10.2196/55297.

[6] C. Fang *et al.*, “Evaluation of an expectation and outcome measurement questionnaire in ankle fracture patients: The Trauma Expectation Factor Trauma Outcomes Measure (TEFTOM) Eurasia study.,” *J. Orthop. Surg. (Hong Kong)*, vol. 28, no. 1, p. 2309499019890140, 2020, doi: 10.1177/2309499019890140.

[7] S. R. Beers *et al.*, “Validity of a pediatric version of the Glasgow Outcome Scale-Extended.,” *J. Neurotrauma*, vol. 29, no. 6, pp. 1126–1139, Apr. 2012, doi: 10.1089/neu.2011.2272.

[8] F. C. M. Reith, R. Van den Brande, A. Synnot, R. Gruen, and A. I. R. Maas, “The reliability of the Glasgow Coma Scale: a systematic review.,” *Intensive Care Med.*, vol. 42, no. 1, pp. 3–15, Jan. 2016, doi: 10.1007/s00134-015-4124-3.

[9] L. Rosenkrantz, N. Schuurman, M. S. Hameed, R. Boniface, and R. Lett, “The Kampala Trauma Score: A 20-year track record,” *J. Trauma Acute Care Surg.*, vol. 92, no. 6, 2022.

[10] J. Damulira *et al.*, “New Trauma Score versus Kampala Trauma Score II in predicting mortality following road traffic crash: a prospective multi-center cohort study.,” *BMC Emerg. Med.*, vol. 24, no. 1, p. 130, Jul. 2024, doi: 10.1186/s12873-024-01048-0.
