# Supplementary material for: Impact of Rural Trauma Team Development Education on Prehospital Time, Referral-to-Dispatch Interval, and Neurological and Musculoskeletal Injury Outcomes: Cluster Randomized Controlled Trial
Source: JMIR Hum Factors. 2026 Apr 20;13:e82591. doi: 10.2196/82591 (PMC13094805; doi:10.2196/82591)
Supplement: Multimedia Appendix 2 [file humanfactors-v13-e82591-s002.docx]

Multimedia Appendix 2: Protocol deviations and implementation fidelity.

| Serial# | Timeline | Description | Protocol deviation, rationale, and effects on the study |
| --- | --- | --- | --- |
| 1 | September 2019 | Protocol registration | This study utilized data from a piloted Motorcycle Trauma Outcome Registry (MOTOR) project. At the time the protocol was submitted, the ethics committee at Uganda National Council for Science and Technology did not require prior registration of non-industry funded academic non‑pharmaceutical proof‑of‑concept projects (policy effective 31 December 2023). As local policy evolved, the investigators pursued registration with the Pan African Clinical Trial Registry. Owing to COVID‑19–related disruptions, registration was only completed after recruitment had begun. This retrospective registration and its justification are disclosed in the abstract and main text. |
| 2 | September 2019 | Inclusion criteria | We had planned to restrict the study to surgery residents, intern doctors, intern nurses, and medical trainees. However, after field observation that 90% of emergency department staff had not undertaken any training specific to emergency care, we included staff involved in care of patients at accident and emergency departments who committed to completing the training for purposes of quality control and implementation fidelity of the study. |
| 3 | March 2020 to March 2021 | Data collection | We had planned to collect the data for the entire twelve study periods, each corresponding to three months, without interruption. However, due to Covid-19 lockdowns which led to inaccessibility of study participants, the study was suspended by the research and ethics committee for a period of one year from March 2020 to March 2021 in accordance with Government regulations to control the spread of Covid-19. Consequently, we conducted the study for 3 years instead of initial planned 4 years. |
| 4 | April 2021 | Training implementation | The training intervention was originally designed as a one-day event for 30 participants, with a trainer-to-trainee ratio of 1:10. However, this plan was changed after the first two study periods due to local Covid-19 regulations and ethical guidelines that restricted attendance to a maximum of 20 participants per training. Thus, when the study resumed in April 2021, the educational activity was extended to two days per training, and the trainer-to-trainee ratio was adjusted to 1:5. The other elements of the training, including data collection methods, variable coding, course delivery, and content, remained unchanged for the remaining ten study periods. |
| 5 | April 2021 to Augst 2023 | Implementation fidelity | We had planned to train 80% of medical trainees and staff affiliated with the accident and emergency departments due to foreseen extreme human constraints arising from understaffing. During the entire study period, eleven staff dropped from the training of which four dropouts were due to leave of absence related to sickness and seven due to failure to get time away from work to complete the training. This was because all hospitals were functioning at low staff capacity ranging from 28 to 48% compared to the minimum sanctioned vacancies approved for the hospital structures by the Ugandan Ministry of Health.  To maintain the 80% coverage, we replaced the eleven dropouts with additional training for medical interns from subsequent intakes. However, since the intervention utilized a team approach to injury care, deploying a five-member trauma team on duty at any single time instead of one trained individual treating one patient, we did not plan to conduct “as-treated analyses” for the cluster-level proportion of trained providers and its association with outcomes to address individual-level exposure misclassification (patient cared for by untrained vs trained providers within the same intervention hospital).  Since the 80% coverage in intervention hospitals meant a 1:4 likelihood that in their continuum of care, a patient may encounter one untrained versus 4 trained providers within a typical five-member trauma team at the same intervention hospital, we believe any biased estimates trend away from the null and this well acknowledged in the study limitations. |
| 6 | September 2023- May 2024 | Protocol Publication | We intended to publish the study protocol before or shortly after data collection began, but because of the Covid-19 pandemic which hit shortly after commencement of the study, the trial was suspended for one year and resumption was only possible after making necessary amendments to meet the local ethical regulations and Ugandan Ministry of Health guidelines regarding social gatherings which included screening of all study participants for Covid-19, adding Covid-19 on the patients’ comorbidity list, protection of trial participants and data collection team with relevant protective gear and trimming the number of course participants from 20 to 15 per training session.  The trial protocol was published with these amendments in May 2024 before data analysis for the main trial, due to the prioritization of Covid-19-related literature for publication, and a general shortage of peer reviewers during the pandemic period. |
| 7 | April 2024 | Data analysis plan | We had planned a possibility of comparing the difference in primary and secondary outcomes using a two-sample t-test but because of skewness of our data despite log transformation, non-parametric two-sample Wilcoxon rank-sum tests and the restricted maximum likelihood (RELM) mixed effects regression models with Satterthwaite adjustment for small sample correction were used to compare the effect sizes for all study outcomes. Also given the study interruption, we validated Hussey and Hughes stratum-by-time predictions of prehospital interval across study periods using cluster robust standard errors in random effects model.  We had planned to report detailed results of subgroup analyses by injury mechanism and severity, and factors associated with all-cause mortality and morbidity based on unfavorable Glasgow Outcome Scale (GOS) and unfavorable Trauma Outcome Measure Score (TOMS) in the main trial but due to manuscript length constraints, we reported these results separately in a MOTOR trial ancillary study cited in the main trial. |
| 8 | April-September 2024 | Data analysis completion | We had planned to complete the data analysis by Spring 2024 and publish results of the main trial by Autumn 2024 but because of competing commitments and timelines amongst the research team, this was only possible by end of 2025. |
| 9 | January 2025 | Exploratory analyses and stratification of data by Covid-19 pandemic status | Since the study was disrupted by Covid-19 pandemic which impacted emergency medical services delivery such as strain on ambulance systems, emergency medical supplies and personnel, we stratified the datasets as before and after pandemic to explore and control for the potential confounding effects of the pandemic on study outcomes such as prehospital interval. However, the key primary and secondary outcomes and their pre-planned methods of analysis remained constant. |
| 10 | December 2025 | Post-hoc power and sample size analyses | We had not planned post-hoc sample size analyses but because we observed the sample for one of the primary outcomes (referral-dispatch interval) was lower than predicted, we performed power and sample analyses to enable future replication studies and to validate the adequacy of the observed sample sizes to achieve effect sizes for the various study outcomes. We found that except for one of the secondary outcomes (TEFS/TOMS), the samples were adequate. |
| 11 | 2019-2025 | Primary and secondary outcomes | This study aimed to assess the effect of rural trauma team development course on both system processes and patient-centered outcomes with focus on motorcycle injuries which are a huge trauma burden in Uganda. Given the immature prehospital care system in Uganda, we felt tailoring the primary outcomes on prehospital and in-hospital system issues would provide more actionable information compared to individual patient outcomes.  Thus, putting into context, the golden hour principle which mandates definitive injury care within one hour after injury and previous studies that had correlated delayed hospital arrival with mortality, the primary outcomes of the study were to compare the (i) prehospital time-from accident scene to emergency department and (ii) referral-dispatch intervals-from referral decision to hospital exit (dispatch).  The secondary outcomes put into context both clinician and patient-centered morbidity outcomes for traumatic brain (TBI) and musculoskeletal (MSK) injuries using Glasgow Outcome Scale (GOS) and Trauma Outcome Measure Scores (TEFS/TOMS) respectively. Thus, the secondary outcomes were to compare (i) 90-day all-cause mortality, (ii) proportion of unfavorable GOS-TBI and (iii) Proportion of unfavorable TEFS/TOMS.  Both primary and secondary outcomes did not change and were reported in the main trial. |
| 12 | 2019-2025 | Tertiary outcomes | We planned to report tertiary outcomes including the effect of the training on provider’s knowledge and the barriers encountered during execution of trauma care for our patient participants to further understand the results of the trial. Due to manuscript length constraints, we reported the results for these tertiary outcomes separately in two ancillary studies cited within the main trial. |
