# Supplementary material for: Impact of Rural Trauma Team Development Education on Prehospital Time, Referral-to-Dispatch Interval, and Neurological and Musculoskeletal Injury Outcomes: Cluster Randomized Controlled Trial
Source: JMIR Hum Factors. 2026 Apr 20;13:e82591. doi: 10.2196/82591 (PMC13094805; doi:10.2196/82591)
Supplement: Multimedia Appendix 3 [file humanfactors-v13-e82591-s003.docx]

Multimedia Appendix 3: Baseline sociodemographic and clinical characteristics of participants.

| Characteristic | Categories | Intervention Group (N=501)  n (%) | | Control Group (N=502)  n (%) | | Overall (N=1003)  n (%) | p-value ^a^ |
| --- | --- | --- | --- | --- | --- | --- | --- |
| General demographic and clinical characteristics across all participants | | | | | | | |
| Age, (Years) | Median (IQR) | 28 (22.0-38.0) | | 28 (22.0-36.0) | | 28 (22.0-37.0) | .83^t^ |
| Sex | |  | |  | |  |  |
|  | Male | 402 (80.2) | | 415 (82.7) | | 817 (81.5) | .32 |
|  | Female | 99 (19.8) | | 87 (17.3) | | 186 (18.5) |  |
| Marital status | |  | |  | |  |  |
|  | Single | 184 (36.7) | | 197 (39.2) | | 381 (38.0) | .30 |
|  | Married | 286 (57.1) | | 285 (56.8) | | 571 (56.9) |  |
|  | Divorced | 21 (4.2) | | 16 (3.2) | | 37 (3.7) |  |
|  | Other | 10 (2.0) | | 4 (0.8) | | 14 (1.4) |  |
| Employment status | |  | |  | |  |  |
|  | Formal paid employment | 33 (6.6) | | 61 (12.2) | | 94 (9.4) | .02 |
|  | Self-employed | 349 (69.7) | | 319 (63.5) | | 668 (66.6) |  |
|  | Unemployed | 25 (5.0) | | 36 (7.2) | | 61 (6.1) |  |
|  | Student | 83 (16.6) | | 76 (15.1) | | 159 (15.9) |  |
|  | Other | 11 (2.2) | | 10 (2.0) | | 21 (2.1) |  |
| Commute distance, (Km) | Median (IQR) | 5.0 (2.0-10.0) | | 5.0 (2.0-10.0) | | 5.0 (2.0-10.0) | .07^t^ |
| Road user category | |  | |  | |  |  |
|  | Passenger | 175 (34.9) | | 153 (30.5) | | 328 (32.7) | .32 |
|  | Pedestrian | 93 (18.6) | | 102 (20.3) | | 195 (19.4) |  |
|  | Motorcyclist | 233 (46.5) | | 247 (49.2) | | 480 (47.9) |  |
| Injury Mechanism | |  | |  | |  |  |
|  | Motorcycle-motorcycle crash | 206 (41.1) | | 225 (44.8) | | 431 (43.0) | .03 |
|  | Motorcycle-pedestrian | 114 (22.7) | | 139 (27.7) | | 253 (25.2) |  |
|  | Motorcycle-car crash | 113 (22.6) | | 87 (17.3) | | 200 (19.9) |  |
|  | Motorcycle-static object | 68 (13.6) | | 51 (10.2) | | 119 (11.9) |  |
| Means of arrival | |  | |  | |  |  |
|  | Ambulance | 96 (19.2) | | 103 (20.5) | | 199 (19.8) | .44 |
|  | Public means (taxi/motorcycle) | 398 (79.4) | | 378 (75.3) | | 776 (77.4) |  |
| Systolic blood pressure at admission, (mmHg) | |  | |  | |  |  |
|  | ≤ 49 | 0 (0.0) | | 2 (0.4) | | 2 (0.2) | .37 |
|  | 50 – 89 | 89 (17.8) | | 90 (17.9) | | 179 (17.8) |  |
|  | > 90 | 412 (82.2) | | 410 (81.7) | | 822 (82.0) |  |
| Respiratory rate at admission (breaths per minute) | |  | |  | |  |  |
|  | ≤ 9 | 4 (0.8) | | 4 (0.8) | | 8 (0.8) | .02 |
|  | 10-29 | 388 (77.4) | | 423 (84.3) | | 811 (80.9) |  |
|  | ≥ 30 | 109 (21.8) | | 75 (14.9) | | 184 (18.3) |  |
| Oxygen circulation at admission | |  | |  | |  |  |
|  | > 90% | 398 (79.4) | | 404 (80.5) | | 802 (80.0) | .68 |
|  | ≤ 90% | 103 (20.6) | | 98 (19.5) | | 201 (20.0) |  |
| Pre-hospital care (first aid) received before arrival) | |  | |  | |  |  |
|  | No | 282 (56.3) | | 262 (52.2) | | 544 (54.2) | .19 |
|  | Yes | 219 (43.7) | | 240 (47.8) | | 459 (45.8) |  |
| Evidence of any chronic medical illness ^e^ | |  | |  | |  |  |
|  | No | 463 (92.4) | | 477 (95.0) | | 940 (93.7) | .09 |
|  | Yes | 38 (7.6) | | 25 (5.0) | | 63 (6.3) |  |
| Multiplicity of serious injuries | |  | |  | |  |  |
|  | > One | 127 (25.3) | | 94 (18.7) | | 221 (22.0) | .02 |
|  | One | 291 (58.1) | | 332 (66.1) | | 623 (62.1) |  |
|  | None | 83 (16.6) | | 76 (15.1) | | 159 (15.9) |  |
| Injury severity score based on Kampala Trauma Score | Median (IQR) | 8 (7-9) | | 8 (7-9) | | 8 (7-9) | .36^t^ |
| Categories of injury severity based on Kampala Trauma Score (KTS) | |  | |  | |  |  |
|  | (9-10) | 233 (46.5) | | 249 (49.6) | | 482 (48.1) | .32 |
|  | (7-8) | 165 (32.9) | | 168 (33.5) | | 333 (33.2) |  |
|  | (≤6) | 103 (20.6) | | 85 (16.9) | | 188 (18.7) |  |
| Injury severity based on Glasgow Coma Scale | Median (IQR) | 14 (12-15) | | 14 (11-15) | | 14 (11-15) | .12^t^ |
| Categories of injury severity based on Glasgow Coma Scale (GCS) | |  | |  | |  |  |
|  | (13-15) | 337 (69.9) | | 301 (64.5) | | 638 (67.2) | .18 |
|  | (9-12) | 100 (20.7) | | 119 (25.5) | | 219 (23.1) |  |
|  | (≤8) | 45 (9.3) | | 47 (10.1) | | 92 (9.7) |  |
| Does the severity or multiplicity of injuries exceed local resources and capacity requiring referral? | |  | |  | |  |  |
|  | No | 158 (31.5) | | 154 (30.7) | | 312 (31.1) | .77 |
|  | Yes | 343 (68.5) | | 348 (69.3) | | 691 (68.9) |  |
| Clinical characteristics of participants with neurological injuries | | | | | | | |
| Reported head trauma (impact) irrespective of symptoms | |  | |  | |  |  |
|  | No | 19 (3.8) | | 35 (7.0) | | 54 (5.4) | .03 |
|  | Yes | 482 (96.2) | | 467 (93.0) | | 949 (94.6) |  |
| Neurological status | |  | |  | |  |  |
|  | Unresponsive | 5 (1.0) | | 4 (0.8) | | 9 (0.9 | .75 |
|  | Responds to pain | 64 (12.8) | | 63 (12.5) | | 127 (12.7) |  |
|  | Responsive to voice | 123 (24.6) | | 138 (27.5) | | 261 (26.0) |  |
|  | Alert | 309 (61.7) | | 297 (59.2) | | 606 (60.4) |  |
| Symptomatic traumatic brain injury present at admission | |  | |  | |  |  |
|  | No | 154 (30.7) | | 150 (29.9) | | 304 (30.3) | .77 |
|  | Yes | 347 (69.3) | | 352 (70.1) | | 699 (69.7) |  |
| Helmet use | | |  | |  |  |  |
|  | No | | 387 (77.2) | | 362 (72.1) | 749 (74.7) | .06 |
|  | Yes | | 114 (22.8) | | 140 (27.9) | 254 (25.3) |  |
| Basis for suspecting traumatic brain injury | | |  | |  |  |  |
|  | High impact (Yes) | | 43 (8.6) | | 35 (7.0) | 78 (7.8) | .34 |
|  | Loss of consciousness (Yes) | | 286 (57.1) | | 271 (54.0) | 557 (55.5) | .32 |
|  | Post-traumatic convulsions (Yes) | | 21 (4.2) | | 15 (3.0) | 36 (3.6) | .31 |
|  | Post-traumatic amnesia (Yes) | | 26 (5.2) | | 27 (5.4) | 53 (5.3) | .89 |
|  | Post-traumatic headache (Yes) | | 150 (29.9) | | 155 (30.9) | 305 (30.4) | .75 |
|  | Alcohol or drug intoxication (Yes) | | 3 (0.6) | | 2 (0.4) | 5 (0.5) | .65^b^ |
|  | Projectile vomiting (Yes) | | 15 (3.0) | | 7 (1.4) | 22 (2.2) | .08 |
|  | Visible injuries above the clavicle (Yes) | | 237 (47.3) | | 239 (47.6) | 476 (47.5) | .92 |
|  | CSF leak (Yes) | | 46 (9.2) | | 44 (8.8) | 90 (9.0) | .82 |
|  | Focal neurological deficit(s) (Yes) | | 65 (13.0) | | 57 (11.4) | 122 (12.2) | .43 |
| Head and brain CT diagnosis | | |  | |  |  |  |
|  | Epidural hematoma | | 93 (18.6) | | 82 (16.3) | 175 (17.4) | .35 |
|  | Subdural hematoma | | 39 (7.8) | | 56 (11.2) | 95 (9.5) | .07 |
|  | Subarachnoid hematoma | | 24 (4.8) | | 20 (4.0) | 44 (4.4) | .53 |
|  | Intraventricular hemorrhage | | 4 (0.8) | | 5 (1.0) | 9 (0.9) | .74^b^ |
|  | Intraparenchymal hemorrhage | | 16 (3.2) | | 20 (4.0) | 36 (3.6) | .50 |
|  | Traumatic axonal/vascular injury | | 9 (1.8) | | 10 (2.0) | 19 (1.9) | .82 |
|  | Cortical contusions | | 31 (6.2) | | 36 (7.2) | 67 (6.7) | .53 |
|  | Skull fracture | | 57 (11.4) | | 40 (8.0) | 97(9.7) | .07 |
|  | Negative CT results | | 114 (22.8) | | 94 (18.7) | 208 (20.7) | .12 |
|  | Others e.g., maxillofacial | | 12 (2.4) | | 15 (3.0) | 27 (2.7) | .56 |
| Neurosurgical intervention | | |  | |  |  |  |
|  | Watchful waiting | | 370 (73.9) | | 372 (74.1) | 742 (74.0) | .17 |
|  | Craniotomy | | 114 (22.8) | | 122 (24.3) | 236 (23.5) |  |
|  | Decompressive craniectomy | | 17 (3.4) | | 8 (1.6) | 25 (2.5) |  |
| Clinical characteristics of participants with musculoskeletal injuries | | | | | | | |
| Musculoskeletal injury present | | |  | |  |  |  |
|  | No | | 101 (20.2) | | 103 (20.5) | 204 (20.3) | .89 |
|  | Yes | | 400 (79.8) | | 399 (79.5) | 799 (79.7) |  |
| Limb fracture present | | |  | |  |  |  |
|  | No | | 367 (73.3) | | 368 (73.3) | 735 (73.3) | .99 |
|  | Yes | | 134 (26.7) | | 134 (26.7) | 268 (26.7) |  |
| Pelvic fracture | | |  | |  |  |  |
|  | No | | 473 (94.4) | | 468 (93.2) | 941 (93.8 | .44 |
|  | Yes | | 28 (5.6) | | 34 (6.8) | 62 (6.2) |  |
| Tibial fracture | | |  | |  |  |  |
|  | No | | 370 (73.9) | | 374 (74.5) | 744 (74.2) | .81 |
|  | Yes | | 131 (26.1) | | 128 (25.5) | 259 (25.8) |  |
| Femur fracture | | |  | |  |  |  |
|  | No | | 487 (97.2) | | 492 (98.0) | 970 (97.6) | .41 |
|  | Yes | | 14 (2.8) | | 10 (2.0) | 24 (2.4) |  |
| Humerus fracture | | |  | |  |  |  |
|  | No | | 500 (99.8) | | 500 (99.6) | 1000 (99.7) | .56 |
|  | Yes | | 1 (0.2) | | 2 (0.4) | 3 (0.3) |  |
| Radius/ulnar fracture |  | |  | |  |  |  |
|  | No | | 496 (99.0) | | 498 (99.2) | 994 (99.1) | .74 |
|  | Yes | | 5 (1.0) | | 4 (0.8) | 9 (0.9) |  |
| Nature of fracture | | |  | |  |  |  |
|  | Open | | 82 (61.2) | | 72 (53.7) | 154 (57.5) | .22 |
|  | Closed | | 52 (38.8) | | 62 (46.3) | 114 (42.5) |  |
| Decision/intent of treatment for the orthopedic or musculoskeletal injury | | |  | |  |  |  |
|  | Conservative ^c^ | | 345 (68.9) | | 363 (72.3) | 708 (70.6) | .23 |
|  | Operative ^d^ | | 156 (31.1) | | 139 (27.7) | 295 (29.5) |  |
| Other associated injuries by body system | | |  | |  |  |  |
|  | Abdomen | | 31 (6.2) | | 24 (4.8) | 55 (5.5) | .33 |
|  | Chest | | 71 (14.2) | | 57 (11.4) | 128 (12.8) | .18 |
|  | Neck | | 41 (8.2) | | 30 (6.0) | 71 (7.1) | .17 |
| Recruitment period | | |  | |  |  |  |
|  | Pre-pandemic | | 82 (16.4) | | 85 (16.9) | 167 (16.7) | .81 |
|  | Post-pandemic | | 419 (83.6) | | 417 (83.1) | 836 (83.4) |  |
| ^a^ Chi-square test of independence except otherwise specified at *P*<.05 level of statistical significance. | | | | | | | |
| ^b^ Fisher’s exact test. | | | | | | | |
| ^c^ Conservative (watchful waiting, physical therapy, casting, braces, splints, pain medication). | | | | | | | |
| ^d^ Operative (open reduction and internal or external fixation or major soft tissue repair requiring theatre). | | | | | | | |
| ^e^ Comorbidity (Diabetes mellitus n=9, Hypertension n=32, HIV/AIDS n=7, ischemic heart disease n=1, Congestive heart failure n=1, Asthma n=8, Chronic obstructive airway disease n=1, Epilepsy n=3, Malnutrition n=1). | | | | | | | |
| ^t^ Two-sample Wilcoxon rank-sum test. | | | | | | | |
| Data are n (%) unless specified otherwise. | | | | | | | |
| Denominators for each treatment group are presented in the top row. | | | | | | | |
| Missing data was excluded from denominators when computing the percentages. | | | | | | | |
| The characteristics of all those included in analyses are demonstrated. | | | | | | | |
