# Supplementary material for: Impact of Rural Trauma Team Development Education on Prehospital Time, Referral-to-Dispatch Interval, and Neurological and Musculoskeletal Injury Outcomes: Cluster Randomized Controlled Trial
Source: JMIR Hum Factors. 2026 Apr 20;13:e82591. doi: 10.2196/82591 (PMC13094805; doi:10.2196/82591)
Supplement: Multimedia Appendix 4 [file humanfactors-v13-e82591-s004.docx]

Multimedia Appendix 4: Adjusted predictions of prehospital time between arms across study periods with confidence intervals.

4A: Showing adjusted predictions of prehospital time across study periods based on Hussey and Hughes stratum-by-time interaction in mixed effects model.


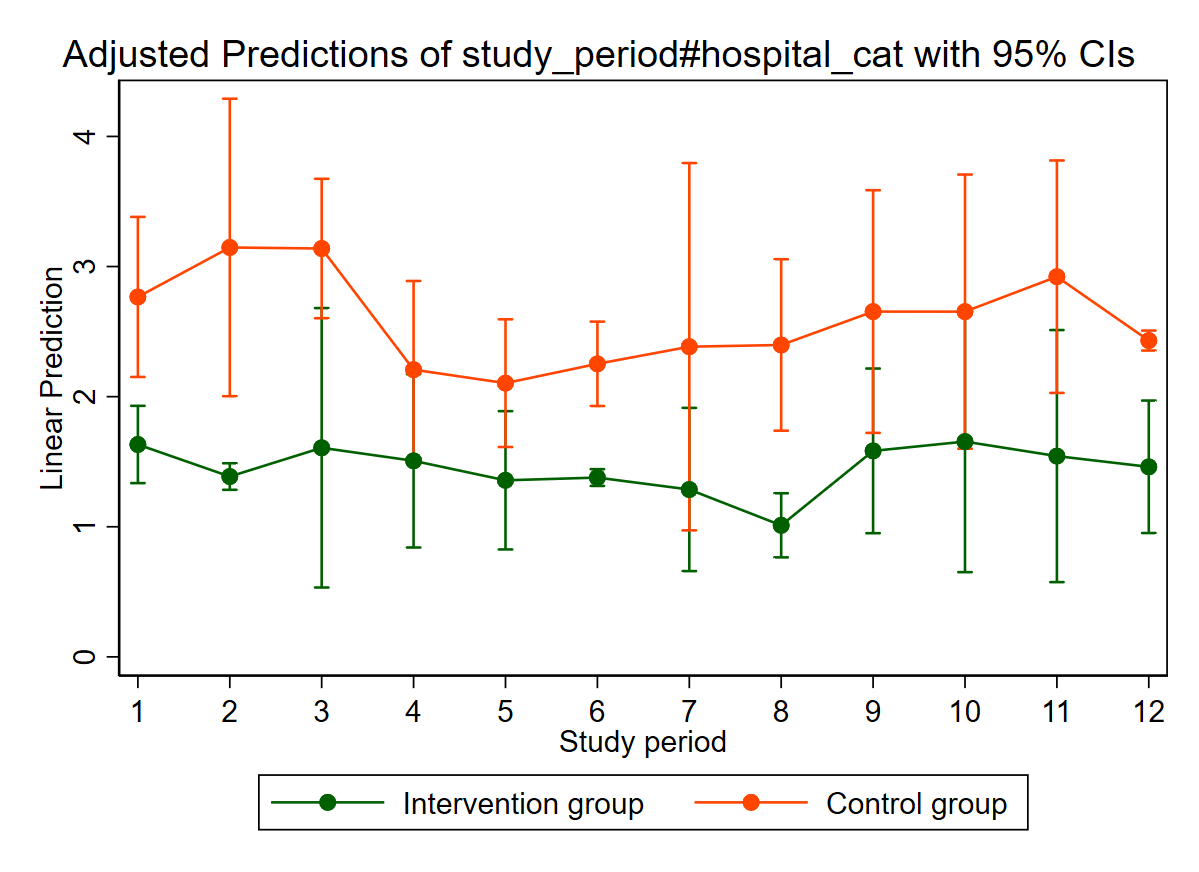


4B: Validation of Hussey and Hughes stratum-by-time predictions of prehospital interval across study periods using cluster robust standard errors in random effects model.
