# Supplementary material for: Impact of Rural Trauma Team Development Education on Prehospital Time, Referral-to-Dispatch Interval, and Neurological and Musculoskeletal Injury Outcomes: Cluster Randomized Controlled Trial
Source: JMIR Hum Factors. 2026 Apr 20;13:e82591. doi: 10.2196/82591 (PMC13094805; doi:10.2196/82591)
Supplement: Multimedia Appendix 5 [file humanfactors-v13-e82591-s005.docx]

Multimedia Appendix 5: Factors associated with delayed prehospital time and referral-dispatch interval of more than one hour.

| Multilevel mixed effects logistic regression analyses of factors associated with delayed prehospital interval of more than one hour. | | | | | | | | | | | | | | | | | | |
| --- | --- | --- | --- | --- | --- | --- | --- | --- | --- | --- | --- | --- | --- | --- | --- | --- | --- | --- |
| Variable | | **Category** | | **Crude Odds Ratio** | | **95% CI** | | | | **P-value** | | **Adjusted Odds Ratio** | | **95% CI** | | | **P-value** | |
| Age | | - | | 1.008 | | .998 | | 1.018 | | .10 | |  | |  | |  |  | |
| Sex | | | |  | |  | |  | |  | |  | |  | |  |  | |
|  | | Female | | Ref | |  | |  | |  | |  | |  | |  |  | |
|  |  | Male | | 1.313 | | .934 | | 1.846 | | .12 | |  | |  | |  |  | |
| Injury mechanism | | | |  | |  | |  | |  | |  | |  | |  |  | |
|  | | Motorcycle-Motorcycle crash | | Ref | |  | |  | |  | |  | |  | |  |  | |
|  |  | Motorcycle-pedestrian crash | | .985 | | .703 | | 1.381 | | .93 | |  | |  | |  |  | |
|  |  | Motorcycle-car crash | | .932 | | .650 | | 1.336 | | .70 | |  | |  | |  |  | |
|  |  | Motorcycle-static object | | 1.523 | | .970 | | 2.391 | | .07 | |  | |  | |  |  | |
| Commute distance from injury to emergency department, (Km) | | | |  | |  | |  | |  | |  | |  | |  |  | |
|  | | <5 Km | |  | |  | |  | |  | | Ref | |  | |  |  | |
|  |  | >5 Km | | 2.019 | | 1.532 | | 2.659 | | <.001 | | 1.850 | | 1.391 | | 2.461 | <.001 | |
| Mode of arrival at emergency department | | | |  | |  | |  | |  | |  | |  | |  |  | |
|  | | By ambulance | | Ref | |  | |  | |  | |  | |  | |  |  | |
|  |  | By public taxi/motorcycle | | .741 | | .525 | | 1.046 | | .09 | |  | |  | |  |  | |
|  |  | By other means (walk-in) | | .633 | | .265 | | 1.517 | | .31 | |  | |  | |  |  | |
| Prehospital care received prior arrival at emergency department? | | | |  | |  | |  | |  | |  | |  | |  |  | |
|  | | Yes | | Ref | |  | |  | |  | | Ref | |  | |  |  | |
|  |  | No | | .369 | | .278 | | .489 | | <.001 | | .408 | | .306 | | .545 | <.001 | |
| Number of serious injuries requiring in-patient admission | | | |  | |  | |  | |  | |  | |  | |  |  | |
|  | | None | | Ref | |  | |  | |  | |  | |  | |  |  | |
|  |  | One | | 1.123 | | .772 | | 1.632 | | .54 | |  | |  | |  |  | |
|  |  | More than one | | 1.607 | | 1.033 | | 2.503 | | .04 | |  | |  | |  |  | |
| Injury severity based on Kampala Trauma Score (KTS) | | | |  | |  | |  | |  | |  | |  | |  |  | |
|  | | Mild (score 9-10) | | Ref | |  | |  | |  | |  | |  | |  |  | |
|  |  | Moderate (score 7-8) | | 1.15 | | .824 | | 1.508 | | .48 | |  | |  | |  |  | |
|  |  | Severe (score ≤6) | | 1.592 | | 1.096 | | 2.313 | | .02 | |  | |  | |  |  | |
| Injury severity based on Glasgow Coma Score (GCS) | | | |  | |  | |  | |  | |  | |  | |  |  | |
|  | | Mild (score 13-15) | | Ref | |  | |  | |  | |  | |  | |  |  | |
|  |  | Moderate (9-12) | | 1.322 | | .946 | | 1.849 | | .10 | |  | |  | |  |  | |
|  |  | Severe (score ≤8) | | 1.987 | | 1.202 | | 3.283 | | .001 | |  | |  | |  |  | |
| Head CT findings | | | |  | |  | |  | |  | |  | |  | |  |  | |
|  | | CT not indicated | | Ref | |  | |  | |  | |  | |  | |  |  | |
|  |  | Negative CT results | | 1.100 | | .760 | | 1.593 | | .61 | |  | |  | |  |  | |
|  |  | Extra-axial lesions | | 1.252 | | .844 | | 1.857 | | .26 | |  | |  | |  |  | |
|  |  | Axial lesions | | 1.403 | | .810 | | 2.427 | | .23 | |  | |  | |  |  | |
|  |  | Skull fractures | | 1.244 | | .742 | | 2.088 | | .41 | |  | |  | |  |  | |
| Referral-dispatch interval | | | |  | |  | |  | |  | |  | |  | |  |  | |
|  | | < 1 hour | | Ref | |  | |  | |  | | Ref | |  | |  |  | |
|  |  | >1 hour | | 1.313 | | .966 | | 1.784 | | .082 | | 1.450 | | 1.041 | | 2.019 | .03 | |
| Neurosurgical treatment | | | |  | |  | |  | |  | |  | |  | |  |  | |
|  | | Watchful waiting | | Ref | |  | |  | |  | | Ref | |  | |  |  | |
|  |  | Craniotomy | | 1.449 | | 1.047 | | 2.005 | | .03 | | 1.489 | | 1.047 | | 2.119 | .03 | |
|  |  | Decompressive craniectomy | | 1.685 | | .709 | | 4.012 | | .24 | | 2.110 | | .852 | | 5.225 | .11 | |
| Mixed effects logistic regression integration method: mvaghermite; Number of observations=1,003; Number of treatment arms=2; Average size per treatment arm=501; CI=Confidence Interval; Residual intraclass correlation (ICC) for intervention vs control arm for adjusted model=0.13, 95% CI (0.02 - 0.52); Akaike’s information criterion (AIC)=1180.16; Bayesian information criterion (BIC)=1214.54 | | | | | | | | | | | | | | | | | | |
|  | | | | | | | | | | | | | | | | | | |
| Mixed effects logistic regression analysis of factors associated with delayed referral-dispatch interval exceeding one hour. | | | | | | | | | | | | | | | | | | |
| Variable | **Category** | | **Crude Odds Ratio** | | **95% CI** | | | | **P-value** | | **Adjusted Odds Ratios** | | **95% CI** | | | | | **P-value** |
| Age | - | | 1.022 | | 1.004 | | 1.040 | | .02 | | 1.024 | | 1.005 | | 1.043 | | | .01 |
| Sex | | |  | |  | |  | |  | |  | |  | |  | | |  |
|  | Female | | Ref | |  | |  | |  | |  | |  | |  | | |  |
|  | Male | | 1.100 | | .618 | | 1.960 | | .75 | |  | |  | |  | | |  |
| Injury mechanism | | |  | |  | |  | |  | |  | |  | |  | | |  |
|  | Motorcycle-motorcycle crash | | 1.289 | | .717 | | 2.314 | | .40 | | Ref | |  | |  | | |  |
|  | Motorcycle-pedestrian crash | | 1.856 | | .957 | | 3.602 | | .07 | | 1.292 | | .689 | | 2.423 | | | .43 |
|  | Motorcycle-car crash | | 2.078 | | .892 | | 4.840 | | .09 | | 2.224 | | 1.112 | | 4.446 | | | .02 |
|  | Motorcycle-static object crash | |  | |  | |  | |  | | 2.096 | | .860 | | 5.107 | | | .10 |
| Commute distance from accident scene to emergency departments, (Km) | | |  | |  | |  | |  | |  | |  | |  | | |  |
|  | <5 Km | | Ref | |  | |  | |  | |  | |  | |  | | |  |
|  | >5Km | | 1.226 | | .760 | | 1.976 | | .40 | |  | |  | |  | | |  |
| Prehospital care received before arrival | | |  | |  | |  | |  | |  | |  | |  | | |  |
|  | Yes | | Ref | |  | |  | |  | |  | |  | |  | | |  |
|  | No | | 1.128 | | .705 | | 1.803 | | .62 | |  | |  | |  | | |  |
| Mode of arrival at emergency department | | |  | |  | |  | |  | |  | |  | |  | | |  |
|  | By ambulance | | Ref | |  | |  | |  | |  | |  | |  | | |  |
|  | By public taxi/motorcycle | | 1.512 | | .878 | | 2.603 | | .14 | |  | |  | |  | | |  |
| Prehospital interval, (Hrs) | | |  | |  | |  | |  | |  | |  | |  | | |  |
|  | <1hr | | Ref | |  | |  | |  | | Ref | |  | |  | | |  |
|  | >1hr | | 1.379 | | .849 | | 2.241 | | .19 | | 1.525 | | .906 | | 2.565 | | | .11 |
| Number of serious injuries requiring hospitalization ^a^ | | |  | |  | |  | |  | |  | |  | |  | | |  |
|  | None | | Ref | |  | |  | |  | |  | |  | |  | | |  |
|  | One | | .321 | | .125 | | .829 | | .02 | |  | |  | |  | | |  |
|  | More than one | | .265 | | .098 | | .718 | | .01 | |  | |  | |  | | |  |
| Head CT results | | |  | |  | |  | |  | |  | |  | |  | | |  |
|  | CT not indicated | | Ref | |  | |  | |  | | Ref | |  | |  | | |  |
|  | Negative CT results | | 40.196 | | 5.355 | | 301.722 | | <.001 | | 44.400 | | 5.885 | | 334.968 | | | <.001 |
|  | Extra-axial lesions | | .681 | | .376 | | 1.235 | | .21 | | .725 | | .395 | | 1.329 | | | .30 |
|  | Axial-lesions | | 1.280 | | .485 | | 3.379 | | .62 | | 1.252 | | .467 | | 3.354 | | | .66 |
|  | Skull fractures | | .536 | | .2651 | | 1.085 | | .08 | | .557 | | .270 | | 1.147 | | | .10 |
| Injury severity based on Kampala Trauma Score (KTS) | | |  | |  | |  | |  | |  | |  | |  | | |  |
|  | Mild (score 9-10) | | Ref | |  | |  | |  | | Ref | |  | |  | | |  |
|  | Moderate (score7-8) | | .414 | | .234 | | .732 | | .002 | | .914 | | .459 | | 1.819 | | | .80 |
|  | Severe (score ≤6) | | .103 | | .177 | | .608 | | <.001 | | .755 | | .313 | | 1.822 | | | .53 |
| Injury severity based on Glasgow Coma Scale (GCS) | | |  | |  | |  | |  | |  | |  | |  | | |  |
|  | Mild (score 13-15) | | Ref | |  | |  | |  | | Ref | |  | |  | | |  |
|  | Moderate (score 9-12) | | .461 | | .270 | | .787 | | .01 | | 1.215 | | .520 | | 2.836 | | | .65 |
|  | Severe (score ≤ 8) | | .480 | | .231 | | .998 | | .049 | | 1.244 | | .416 | | 3.720 | | | .70 |
| Neurosurgical management | | |  | |  | |  | |  | |  | |  | |  | | |  |
|  | Watchful waiting | | Ref | |  | |  | |  | | (Ref) | |  | |  | | |  |
|  | Craniotomy | | .414 | | .249 | | .687 | | .001 | | 1.252 | | .512 | |  | | | .62 |
|  | Decompressive craniectomy | | .325 | | .111 | | .946 | | .04 | | .676 | | .164 | |  | | | .59 |
| Mixed effects logistic regression were performed using melogit command and mvaghermite integration method. Statistical significance level at P<.05; Number of observations=979; Number of treatment arms=2; Average size per treatment arm=501; CI=Confidence Interval; Residual intraclass correlation (ICC) for intervention vs control arm for adjusted model=0.34, 95% CI (0.06 - 0.80); Akaike’s information criterion (AIC)=438.25; Bayesian information criterion (BIC)=487.12; ^a^ Serious injury refers to one that warranty admission on its own merit. Physicians’ estimation of number of serious injuries correlates with Abbreviated Injury Severity Score (Gardner A, Forson PK, Oduro G, Stewart B, Dike N, Glover P, et al. Diagnostic accuracy of the Kampala Trauma Score using Estimated Abbreviated Injury Scale scores and physician opinion. *Injury.* 2017;48(1):177–183). | | | | | | | | | | | | | | | | | | |
