# Supplementary material for: Impact of Rural Trauma Team Development Education on Prehospital Time, Referral-to-Dispatch Interval, and Neurological and Musculoskeletal Injury Outcomes: Cluster Randomized Controlled Trial
Source: JMIR Hum Factors. 2026 Apr 20;13:e82591. doi: 10.2196/82591 (PMC13094805; doi:10.2196/82591)
Supplement: Multimedia Appendix 7 [file humanfactors-v13-e82591-s007.docx]

Multimedia Appendix 7: Stratified Cox regression analysis of determinants of time-to-event (death).

| Variable | Category | Hazard Ratio (HR)^a^ | 95% Confidence Interval | P-value ^b^ |
| --- | --- | --- | --- | --- |
| Prehospital care received before arrival to emergency department | |  |  |  |
|  | No (ref) |  |  |  |
|  | Yes | 0.96 | 0.58 – 1.57 | .87 |
| Referral decision to dispatch interval | |  |  |  |
|  | Less than one hours (ref) |  |  |  |
|  | More than one hour | 1.25 | 0.65 – 2.41 | .51 |
| Kampala Trauma Score (KTS) | |  |  |  |
|  | Mild injury  (score 9-10) (ref) |  |  |  |
|  | Moderate injury (score 7-8) | 1.11 | 0.30 – 4.17 | .87 |
|  | Severe injury (score 6 or less) | 1.69 | 0.45 – 6.37 | .44 |
| Glasgow comma score (GCS) | |  |  |  |
|  | Mild injury  (score 13-15) (ref) |  |  |  |
|  | Moderate injury (score 9-12) | 0.97 | 0.42 – 2.24 | .94 |
|  | Severe injury (score 8 or less) | 1.614 | 0.68 – 3.84 | .28 |
| Diagnosis based on head CT | |  |  |  |
|  | Extra-axial haematomas (ref) |  |  |  |
|  | Intra-axial haematomas | 0.96 | 0.55 – 1.67 | .87 |
| Neurosurgical intervention | |  |  |  |
|  | Watchful waiting (ref) |  |  |  |
|  | Craniotomy | 0.35 | 0.17 – 0.73 | .01 |
|  | Craniectomy | 0.459 |  | .09 |
| Level of statistical significance at *P*<.05. ^a^ Cox regression models was approximated with Breslow method for ties; stratified by treatment allocation (intervention vs. control). ^b^ The Schoenfeld residuals test for the proportional hazards’ assumption from this stratified Cox regression model indicated that all p-values were greater than .05 for the variables included, with a global test p-value of .49. This suggests that the assumption of constant hazard ratios between groups was met. | | | | |
