# Supplementary figures and images for: Impact of Rural Trauma Team Development Education on Prehospital Time, Referral-to-Dispatch Interval, and Neurological and Musculoskeletal Injury Outcomes: Cluster Randomized Controlled Trial
Source: JMIR Hum Factors. 2026 Apr 20;13:e82591. doi: 10.2196/82591 (PMC13094805; doi:10.2196/82591)

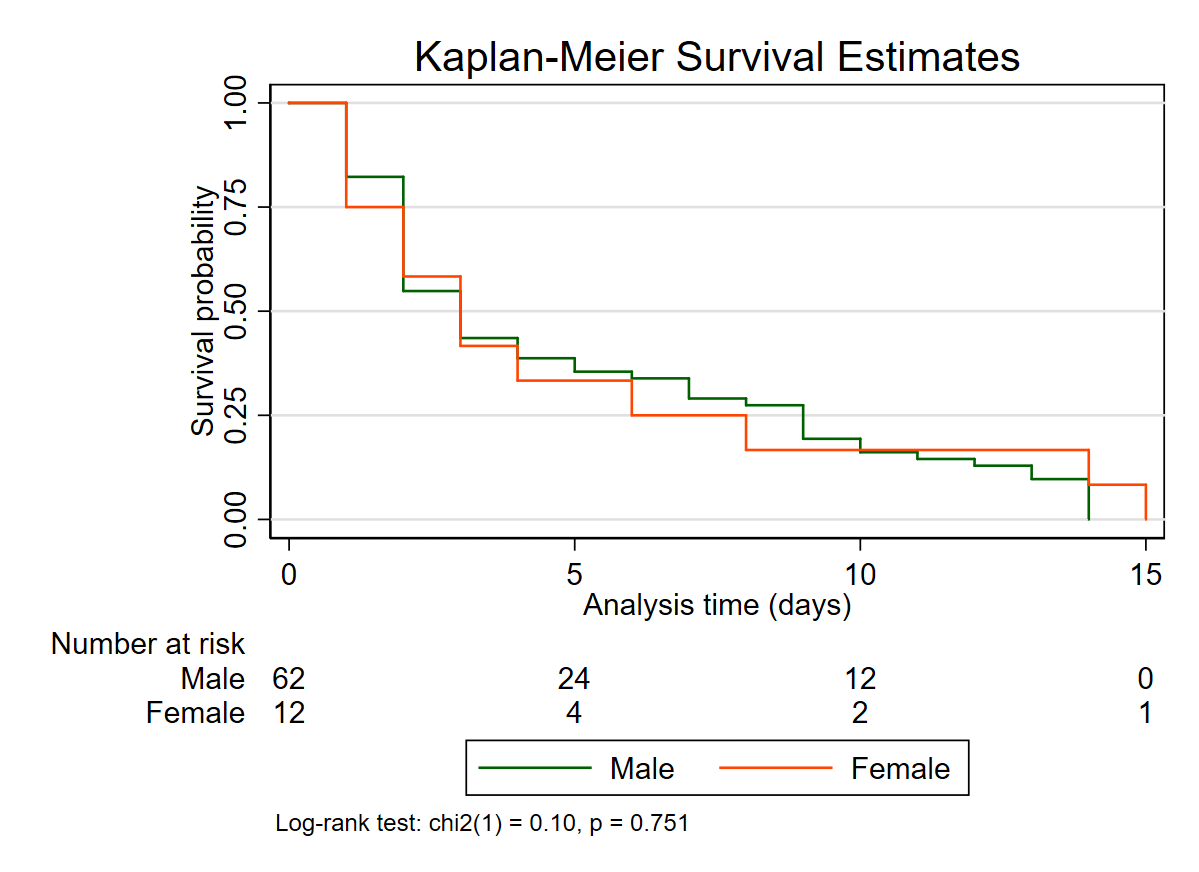


Multimedia Appendix 8: Subgroup analysis of survival time in days stratified by sex.

Supplement: Multimedia Appendix 8 [file humanfactors-v13-e82591-s008.docx]

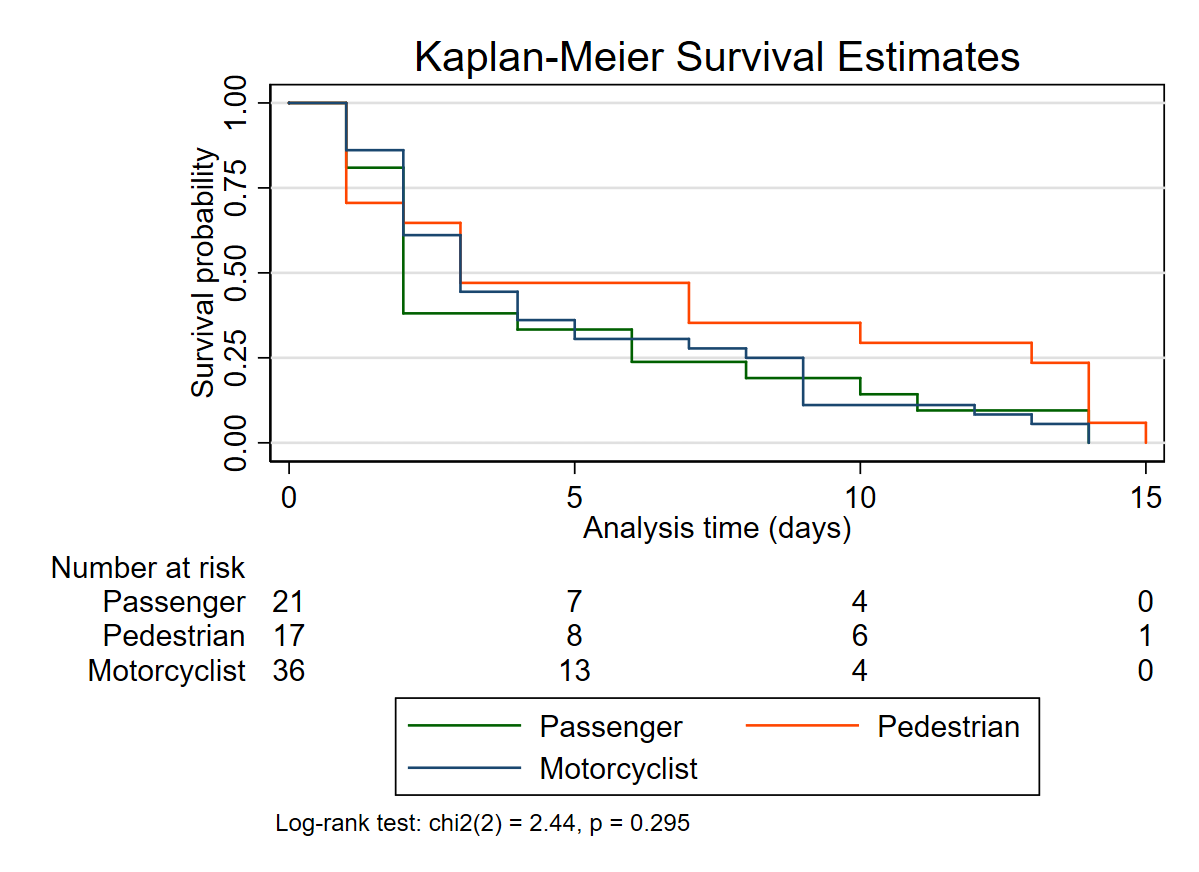


Multimedia Appendix 9: Subgroup analysis of survival time in days by road user category.

Supplement: Multimedia Appendix 9 [file humanfactors-v13-e82591-s009.docx]
