# Supplementary material for: Impact of Rural Trauma Team Development Education on Prehospital Time, Referral-to-Dispatch Interval, and Neurological and Musculoskeletal Injury Outcomes: Cluster Randomized Controlled Trial
Source: JMIR Hum Factors. 2026 Apr 20;13:e82591. doi: 10.2196/82591 (PMC13094805; doi:10.2196/82591)
Supplement: Multimedia Appendix 10 [file humanfactors-v13-e82591-s010.docx]

Multimedia Appendix 10: Comparison of Glasgow Outcome Score at 90 days between treatment groups.

| GOS | Variable | Intervention n (%) | Control n (%) | Overall |
| --- | --- | --- | --- | --- |
| 1 | Death (clinically confirmed death) | 24 (5.2) ^a^ | 58 (13.5) ^b^ | 82 (9.2) |
| 2 | Persistent vegetative state (severe damage with prolonged state of unresponsiveness and lack of higher mental function) | 1 (0.2) | 1 (0.2) | 2 (0.2) |
| 3 | Severe disability (severe injury with permanent need for help with daily living) | 17 (3.7) | 26 (6.0) | 43 (4.8) |
| 4 | Moderate disability (no need for assistance in everyday life, employment is possible but may require special equipment) | 123 (26.9) | 101 (23.5) | 224 (25.3) |
| 5 | Good recovery (minimal injury with minor neurological and psychological deficits, patient is independent and employable | 292 (63.9) ^c^ | 244 (56.7) ^d^ | 536 (60.4) |
|  | Total | 457 | 430 | 887 |
| ^a, b, c, d^ Denotes subsets whose column proportions differ significantly from each other at the 95% confidence interval level. | | | | |
