# Supplementary material for: Impact of Rural Trauma Team Development Education on Prehospital Time, Referral-to-Dispatch Interval, and Neurological and Musculoskeletal Injury Outcomes: Cluster Randomized Controlled Trial
Source: JMIR Hum Factors. 2026 Apr 20;13:e82591. doi: 10.2196/82591 (PMC13094805; doi:10.2196/82591)
Supplement: Multimedia Appendix 11 [file humanfactors-v13-e82591-s011.docx]

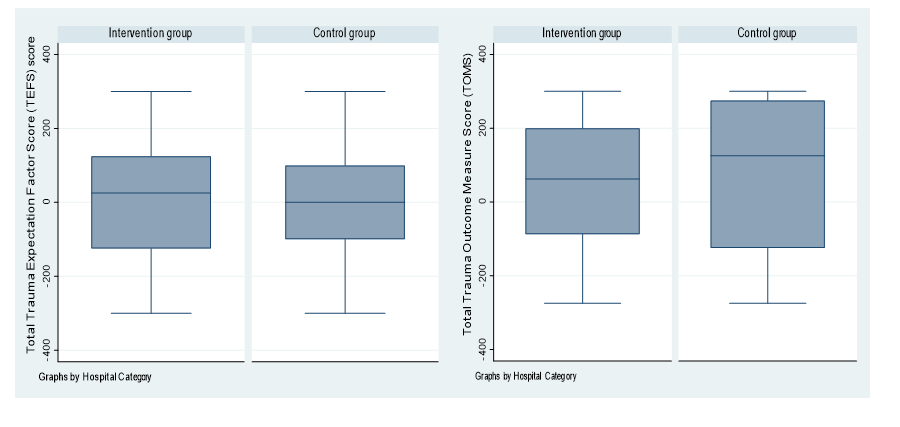


Multimedia Appendix 11: Subgroup analysis of TEFS and TOMS for participants with tibial fractures.
