# Supplementary material for: Impact of Rural Trauma Team Development Education on Prehospital Time, Referral-to-Dispatch Interval, and Neurological and Musculoskeletal Injury Outcomes: Cluster Randomized Controlled Trial
Source: JMIR Hum Factors. 2026 Apr 20;13:e82591. doi: 10.2196/82591 (PMC13094805; doi:10.2196/82591)
Supplement: Multimedia Appendix 12 [file humanfactors-v13-e82591-s012.docx]

Multimedia Appendix 12: Trauma Expectation Factor Scores (TEFS) and Trauma Outcome Measure Scores (TOMS) across study periods.
