# Supplementary material for: Impact of Rural Trauma Team Development Education on Prehospital Time, Referral-to-Dispatch Interval, and Neurological and Musculoskeletal Injury Outcomes: Cluster Randomized Controlled Trial
Source: JMIR Hum Factors. 2026 Apr 20;13:e82591. doi: 10.2196/82591 (PMC13094805; doi:10.2196/82591)
Supplement: Multimedia Appendix 13 [file humanfactors-v13-e82591-s013.docx]

Multimedia Appendix 13: Residual estimates from per-protocol comparison of primary and secondary outcomes after small sample correction.

| **Outcome variable (number of observations)** | **Estimate** | **95% Confidence interval (lower, upper)** | **Residual standard error** |
| --- | --- | --- | --- |
| Primary outcome 1: prehospital interval (n=1,003) | 1.72 | 1.58, 1.88 | .08 |
| Primary outcome 2: referral-dispatch interval in hours (n=691) | 1.19 | 1.07, 1.33 | .07 |
| Secondary outcome 1: percentage of 90-day mortality (n=887) | 0.92 | 0.84, 1.00 | .04 |
| Secondary outcome 2: percentage of Unfavorable Glasgow Outcome Scale-GOS (n=887) | 0.12 | 0.11, 0.13 | .01 |
| Secondary outcome 3: percentage of unfavorable Trauma Outcome Measure Score-TOMS (n=637) | 0.19 | 0.17, 0.21 | .01 |
| Estimates for random effects parameters were computed in mixed effects restricted maximum likelihood (RELM) regression models using Satterthwaite method of adjustment of degrees of freedom for small sample correction. | | | |
| Number of study arms: 2 (intervention vs control) | | | |
| Total number of clusters: 6 | | | |
| Number of clusters per treatment arm: 3 | | | |
| Number of discrete time decay periods: 12 | | | |
