# Supplementary material for: Impact of Rural Trauma Team Development Education on Prehospital Time, Referral-to-Dispatch Interval, and Neurological and Musculoskeletal Injury Outcomes: Cluster Randomized Controlled Trial
Source: JMIR Hum Factors. 2026 Apr 20;13:e82591. doi: 10.2196/82591 (PMC13094805; doi:10.2196/82591)
Supplement: Multimedia Appendix 14 [file humanfactors-v13-e82591-s014.docx]

Multimedia Appendix 13: Sensitivity analyses of injury outcomes under different case scenarios.

| Variable | Scenario | Base Case Scenario ^a^ | | | Worst Case Scenario ^b^ | | | Best Case Scenario ^c^ | | |
| --- | --- | --- | --- | --- | --- | --- | --- | --- | --- | --- |
|  | Category | Intervention | Control | p-value | Intervention | Control | p-value | Intervention | Control | p-value |
| Prehospital Time (hrs) | Median (IQR) | 1 (0.5-2.0) | 2 (1.50-4.00) | <.001 | 1 (0.5-2.0) | 2 (1.5-4.0) | <.001 | 1(0.5-2.0) | 2 (1.5-4.0) | <.001 |
| Referral Exit Interval (hrs) | Median (IQR) | 2 (1.3-2.8) | 4 (2.5-4.1) | <.001 | 2 (1.3-2.8) | 3.6 (2.5-4.1) | <.001 | 2 (1.3-2.8) | 3.6 (2.5-4.1) | <.001 |
| 90-day Injury Outcome | |  |  |  |  |  |  |  |  |  |
|  | Survived (Ref) | 433 (94.8) | 372 (86.5) | <.001 | 433 (86.4) | 444 (88.5) | .34 | 477 (95.2) | 372 (74.1) | <.001 |
|  | Died | 24 (5.3) | 58 (13.5) |  | 68 (13.6) | 58 (11.6) |  | 24 (4.8) | 130 (25.9) |  |
| 90-day Glasgow Outcome Scale (GOS) | |  |  |  |  |  |  |  |  |  |
|  | Favourable (GOS 4-5) (Ref) | 415 (90.8) | 343 (79.8) | <.001 | 415 (85.9) | 383 (81.5) | .06 | 441 (91.3) | 343 (73.0) | <.001 |
|  | Unfavourable (GOS 1-3) | 42 (9.2) | 87 (20.2) |  | 68 (14.1) | 87 (18.5) |  | 42 (8.7) | 127 (27.0) |  |
| 90-day Trauma Outcome Measure Score (TOMS) | |  |  |  |  |  |  |  |  |  |
|  | Favourable ^d^ (Ref) | 262 (76.0) | 216 (74.0) | .56 | 316 (78.6) | 320 (80.6) | .48 | 316 (78.6) | 320 (80.6) | .48 |
|  | Unfavourable ^e^ | 83 (24.0) | 76 (26.0) |  | 86 (21.4) | 77 (19.4) |  | 86 (21.4) | 77 (19.4) |  |
| ^a^ Base case scenario: Complete case analysis, assuming all missing data were missing completely at random. | | | | | | | | | | |
| ^b^ Worst case scenario: Assuming all participants lost to follow-up in the intervention group experienced the worst trauma outcomes (e.g., died or had unfavourable outcomes), while all those with missing data in the control group had favourable outcomes (e.g., survived). | | | | | | | | | | |
| ^c^ Best case scenario: Assuming all participants with missing data in the intervention group survived with favourable outcomes, while those in the control group died or had unfavourable outcomes. | | | | | | | | | | |
| ^d^ (Trauma outcome measure score at 90-days equal or exceed trauma expectation factor score at baseline). | | | | | | | | | | |
| ^e^ (Trauma outcome measure score at 90-days less than trauma expectation factor score at baseline). | | | | | | | | | | |
| Data are n (%) unless specified otherwise at *P* < .05 level of statistical significance. | | | | | | | | | | |
