# Supplementary material for: Impact of Rural Trauma Team Development Education on Prehospital Time, Referral-to-Dispatch Interval, and Neurological and Musculoskeletal Injury Outcomes: Cluster Randomized Controlled Trial
Source: JMIR Hum Factors. 2026 Apr 20;13:e82591. doi: 10.2196/82591 (PMC13094805; doi:10.2196/82591)
Supplement: Multimedia Appendix 15 [file humanfactors-v13-e82591-s015.docx]

| **Imputed variables with loss to follow-up (missing data)** | **Observations per (*m*)** | | | **(Complete plus incomplete)** |
| --- | --- | --- | --- | --- |
|  | **Complete, n (%)** | **Incomplete, n (%)** | **Imputed, n (%)** | **Total** |
| 90-day injury outcome (mortality) | 887 (88.4) | 116 (11.6) | 116 (11.6) | 1003 |
| TEFS^a^ or TOMS^b^ | 1000 (90.7) | 3 (0.3) | 3 (0.3) | 1003 |
| GCS^c^ | 949 (94.7) | 54 (5.3) | 54 (5.3) | 1003 |
| GOS^d^ | 887 (88.4) | 116 (11.6) | 116 (11.6) | 1003 |
| Imputed is the minimum across “*m*” of the number of filled-in observations. | | | | |
| Imputation method: Multiple imputation by chained equation (MICE). | | | | |
| Number of imputations (*m*): 20 | | | | |
| Assumption: Missing at random or completely missing at random. | | | | |
| ^a^TEFS: Trauma Outcome Measure Score. | | | | |
| ^b^TOMS: Trauma Expectation Factor Score. | | | | |
| ^c^GCS: Glasgow Coma Scale. | | | | |
| ^d^GOS: Glasgow Outcome Scale. | | | | |
