# Supplementary material for: Impact of Rural Trauma Team Development Education on Prehospital Time, Referral-to-Dispatch Interval, and Neurological and Musculoskeletal Injury Outcomes: Cluster Randomized Controlled Trial
Source: JMIR Hum Factors. 2026 Apr 20;13:e82591. doi: 10.2196/82591 (PMC13094805; doi:10.2196/82591)
Supplement: Multimedia Appendix 16 [file humanfactors-v13-e82591-s016.docx]

| **Outcome variable (number of observations)** | **Estimate (95% CI)** | **Residual SE** |
| --- | --- | --- |
| Primary outcome 1: prehospital interval (hours; n=1003) | 1.72 (1.57-1.88) | .08 |
| Primary outcome 2: referral-dispatch interval (hours; n=691) | 1.18 (1.06-1.31) | .06 |
| Secondary outcome 1: percentage of 90-day mortality (%; n=887) | 0.92 (0.84-1.00) | .04 |
| Secondary outcome 2: percentage of unfavorable Glasgow Outcome Scale (GOS, %; n=1003) | 0.12 (0.11-0.13) | .01 |
| Secondary outcome 3: Percentage of unfavorable Trauma Outcome Measure Score (TOMS, %; n=637) | 0.19 (0.17-0.21) | .01 |
