# Supplementary material for: Impact of Rural Trauma Team Development Education on Prehospital Time, Referral-to-Dispatch Interval, and Neurological and Musculoskeletal Injury Outcomes: Cluster Randomized Controlled Trial
Source: JMIR Hum Factors. 2026 Apr 20;13:e82591. doi: 10.2196/82591 (PMC13094805; doi:10.2196/82591)
Supplement: Multimedia Appendix 17 [file humanfactors-v13-e82591-s017.docx]

Multimedia appendix 14: Post-hoc power and sample size analyses for various study outcomes.

Using R shiny CRT application [1], [2], we performed post hoc power analyses to validate the adequacy of the observed sample sizes to achieve effect sizes for the various study outcomes and to permit future replication studies assuming our various case scenarios.

**Scenario A:** We established a targeted power of 80% based on assumptions made regarding the primary outcome, specifically the prehospital interval, prior to the conduct of the study. The assumptions included parallel trial design, t-distribution, a mean difference in prehospital time of 1.02 hours, a pooled standard deviation of 1.64 (derived from existing literature), a significance level of 0.05, a coefficient of variation of 0.5, an intra-cluster correlation coefficient (ICC) of 0.02 with a 95% confidence interval of [0.01, 0.05] and a cluster autocorrelation coefficient of 0.8. In the context of a two-arm parallel cluster randomized trial encompassing twelve discrete study periods, the required average cluster size per period was determined to be ten, with three clusters per arm. Consequently, the average total sample size per cluster for the entire 12 periods amounted to 120 participants (see Fig. A).

Fig A: Number of required clusters and average cluster size to achieve power of 80% based on assumed scenario for prehospital interval.

**Scenario B:** After conducting the study, a power of 80% was achieved for the first primary outcome, specifically the prehospital interval. This was based on an observed intra-cluster correlation coefficient (ICC) of 0.01, with a 95% confidence interval of [0.00, 0.06]. The analysis revealed a mean difference of 1.14 hours and an observed pooled standard deviation of 1.44, at a significance level of 0.05. Additionally, at a coefficient of variation of 0.5, and a cluster autocorrelation coefficient of 0.8, an average cluster size of fourteen participants per period over 12 discrete periods was observed, with three clusters per arm. The observed average cluster size for the entire twelve study periods was 167, with a confidence interval of [141 - 189], meeting the initial target of 120 participants (see Fig. B).

Fig B: Number of required clusters and average cluster size for power of 80% based on observed scenario for prehospital interval.

**Scenario C:** Upon the conclusion of the study, a power of 80% was achieved for the second primary outcome, the referral-dispatch interval, under the assumption of a significance level of 0.05, a coefficient of variation of 0.5, and a cluster autocorrelation coefficient of 0.8. This achievement was based on observed study parameters, including an intra-cluster correlation coefficient (ICC) of 0.01 (95% CI: [0.00, 0.08]), a mean difference of 1.41 hours and a pooled standard deviation of 1.30. The study encompassed three clusters per arm with a total of twelve discrete decay periods, an observed average cluster size of ten participants per period and a total average cluster size of 115 participants (95% CI: [80 - 135]) for the entire duration.

Fig C: Number of required clusters and average cluster size for power of 80% based on observed scenario for referral-dispatch interval.

**Scenario D:** As demonstrated in Fig. D, a power of 80% for the secondary outcome, specifically 90-day mortality, was achieved based on an observed base intra-cluster correlation coefficient (ICC) of 0.01 (95% CI: [0.00, 0.05]). This power was further supported by a mortality proportion of 0.052 in the intervention group and 0.136 in the control group. The average cluster size was thirteen participants per period, resulting in a total cluster size of 147 participants (95% CI: [119 - 181]) across the twelve study periods, with three clusters per arm. These calculations were made under the assumptions of a significance level of 0.05, a coefficient of variation of 0.5, and a cluster autocorrelation coefficient of 0.8. However, future studies that assume an upper ICC of 0.05 and keep other factors constant would necessitate a minimum of six clusters per arm to achieve a study power of 80% (Fig D).

Fig D: Number of required clusters and average cluster size for power of 80% based on observed scenario for 90-day mortality.

**Scenario E:** We observed that a study power of 80% was achieved for dichotomized secondary outcomes related to the proportion of unfavorable Glasgow Outcome Scale (GOS). This assertion was based on an observed base intra-cluster correlation coefficient (ICC) of 0.01 (95% CI: [0.00, 0.07]), a proportion of unfavorable GOS outcomes of 0.091 in the intervention group and 0.205 in the control group, with an average cluster size of 13 participants per period, leading to a total cluster size of 147 participants (95% CI: [119 - 181]) across the 12 study periods with three clusters per arm. These findings were derived under the assumptions of a significance level of 0.05, a coefficient of variation of 0.5, and a cluster autocorrelation coefficient of 0.8. Notably, if the upper ICC is assumed to be 0.07 while maintaining other assumptions, an estimate of six clusters per arm would be required to achieve a study power of 80% in future studies (See Fig E).

Fig E: Number of required clusters and average cluster size for power of 80% based on observed scenario for proportion of unfavorable Glasgow Outcome Scale (GOS).

**Scenario F:** We determined that achieving a power of 80% for secondary outcomes related to the Trauma Outcome Measure Score (TOMS) for musculoskeletal injuries was not feasible. To attain this power, future studies assuming our observed base intra-cluster correlation coefficient (ICC) of 0.01 (95% CI: [0.00, 0.10]), a proportion of unfavorable TOMS of 0.248 in the intervention group and 0.266 in the control group, a cluster correlation coefficient of 0.8, and a coefficient of variation of 0.5 at a significance level of 0.05 would require an estimated 100 clusters per arm with an average cluster size of 10 per period. Alternatively, assuming a lower ICC of 0.001 would necessitate sixty clusters per arm with a cluster size of fifty per period, or an upper ICC of 0.10 would require 430 clusters per arm with a minimum cluster size of fifty participants (see Fig. E). However, these targets may be challenging to achieve in low- and middle-income countries (LMIC) settings due to resource allocation difficulties.

Fig F: Ideal number of required clusters and average cluster size for power of 80% based on observed scenario for proportion of unfavorable Trauma Outcome Measure Score (TOMS).

References:

[1] R. Hooper and L. Bourke, “Cluster randomised trials with repeated cross sections: alternatives to parallel group designs,” *BMJ*, vol. 350, 2015, doi: 10.1136/bmj.h2925.

[2] K. Hemming, J. Kasza, R. Hooper, A. Forbes, and M. Taljaard, “A tutorial on sample size calculation for multiple-period cluster randomized parallel, cross-over and stepped-wedge trials using the Shiny CRT Calculator,” *Int. J. Epidemiol.*, vol. 49, no. 3, pp. 979–995, 2020, doi: 10.1093/ije/dyz237.
