# Supplementary material for: Impact of Rural Trauma Team Development Education on Prehospital Time, Referral-to-Dispatch Interval, and Neurological and Musculoskeletal Injury Outcomes: Cluster Randomized Controlled Trial
Source: JMIR Hum Factors. 2026 Apr 20;13:e82591. doi: 10.2196/82591 (PMC13094805; doi:10.2196/82591)
Supplement: Multimedia Appendix 18 [file humanfactors-v13-e82591-s018.docx]

Multimedia Appendix 15: Reflexivity statement regarding collaborative partnerships.

| Items^[1]^ | Question | Answer |
| --- | --- | --- |
| Study conceptualization | |  |
|  | How does this study address local research and policy priorities? | This study addressed rural trauma training and coordination which are critical issues in Uganda due to its high injury burden and inadequate human resources for health. |
|  | How were local researchers involved in the study? | The PI was a local researcher who leveraged locally available expertise to offer the rural trauma team development training intervention. The local research team assisted with necessary ethical, logistical, and administrative clearances. |
| Research management | How has funding been used to support the local research team? | HIC partners hosted LMIC (PI) through a funded doctoral researcher position at the University of Turku (Finland) to develop local research capacity. The Centre for Health Equity in Surgery and Anesthesia (CHESA) at University of California San Francisco (USA) provided a travel grant to PI to facilitate dissemination of results and offered the PI a 2-year fellowship in global surgery and perioperative medicine. |
| Data acquisition and analysis | |  |
|  | How are research staff who conducted data collection acknowledged? | Research staff who met the authorship criteria detailed in the international committee of medical journal editors (ICMJE) guidelines were included as authors whereas those who did not meet these criteria but made substantial contributions are acknowledged in the acknowledgement section. |
|  | How have members of the research partnership been provided with access to the study data? | The data collection team was issued with login access to the raw data in REDCap and the final dataset is accessible to all partners through unrestricted publishing. |
|  | How was data used to develop analytical skills within the partnership? | LMIC PI developed a locally contextualized data collection tool. HIC partners provided working space and software to LMIC PI to develop capacity for data analysis. |
| Data interpretation | How have research partners collaborated in interpreting the data? | LMIC PI analyzed and interpreted the data with visualization and cross-validation support from internal and external biotechnicians from HICs. |
| Drafting and revising for intellectual content | |  |
|  | How were research partners supported to develop writing skills? | LMIC PI was supported to attend academic writing courses at the University of Turku in Finland. |
|  | How will research products be shared to address local needs? | The study findings were accepted in form of abstract for oral presentation to stakeholders under the theme “equitable and sustainable strategies for injury and violence prevention) during WHO safety 2024 (world conference on injury prevention and safety promotion 2-4 September 2024 in New Delhi, India).  Local regional dissemination was planned at the 16^th^ WHO safety 2026 world conference on prevention and safety promotion during September 2 - 4 in Cape town South Africa, and at the 20^th^ annual surgical scientific conference of the Association of Surgeons of Uganda during 25 - 27 March 2026. |
| Authorship | |  |
|  | How are the leadership, contribution, and ownership of this work by LMIC researchers recognized within the authorship? | Authorship was based on the ICMJE criteria. LMIC PI is the first author and HIC supervisors are the last authors. |
|  | How have early career researchers across partnership been included within the authorship teem? | Early career researchers whose contributions met the inclusion criteria were included as authors. |
|  | How has gender balance been addressed within the authorship? | The authorship was based on ICJME guidelines as per trial protocol without gender bias. |
| Training | How has the project contributed to training LMIC researchers? | The project is part of the LMIC PI’s body of work for a doctoral degree. |
| Infrastructure | How has project contributed to improvements in local infrastructure? | We trained a total of five hundred rural trauma care frontliners during this project to build capacity for injury care in LMICs. Further, we piloted a motorcycle trauma outcome (MOTOR) registry in parallel to the trial. |
| Governance | What safeguarding procedures were used to protect local study participants and researchers? | The study was conducted in accordance with the Uganda National Council for Science and Technology guidelines on human subjects as research participants. |
| [1] B. Morton *et al.*, “Consensus statement on measures to promote equitable authorship in the publication of research from international partnerships.,” *Anaesthesia*, vol. 77, no. 3, pp. 264–276, Mar. 2022, doi: 10.1111/anae.15597. | | |
